# Safety outcomes of statin vs non-statin lipid-lowering interventions in patients with prior statin-associated muscle symptoms: A systematic review and meta-analysis

**DOI:** 10.1371/journal.pone.0338575

**Published:** 2025-12-11

**Authors:** Philipp Stefan Aebi, Fanny Villoz, Jonas Bührer, Christina Lyko, Nazanin Abolhassani, Cinzia Del Giovane, Baris Gencer, Nicolas Rodondi, Manuel R. Blum

**Affiliations:** 1 Institute of Primary Health Care (BIHAM), University of Bern, Bern, Switzerland; 2 Department of General Internal Medicine, Inselspital, Bern University Hospital, University of Bern, Bern, Switzerland; 3 Centre médico-chirurgical de l’obésité, HÔPITAL RIVIERA-CHABLAIS, Route des Tilles 6A, Rennaz, Switzerland; 4 Service of Cardiology, Lausanne University Hospital (CHUV), Lausanne, Switzerland; 5 Spital fmi AG, Weissenaustrasse 27, Unterseen, Switzerland; Universitas Muhammadiyah Aceh, INDONESIA

## Abstract

**Background:**

Statin-associated muscle symptoms (SAMS) are an obstacle in the prevention of cardiovascular events. A systematic assessment of the evidence of interventions in the setting of SAMS is lacking.

**Objective:**

To assess the evidence of strategies of statin-based vs non-statin based therapies in patients with a history of SAMS.

**Methods:**

MEDLINE, EMBASE, Cochrane Central Register of Controlled Clinical Trials, Scopus, Clinicaltrials.gov and Proquest databases were searched from inception up to February 2024.We included randomized controlled trials (RCTs) and non-randomized studies involving patients with history of prior SAMS, comparing statin-based therapy to a comparator. We followed the PRISMA guideline with multiple authors involved at each stage. A random-effect model was used in the meta-analysis. We defined the primary outcome as incidence of muscle symptoms. The secondary outcomes were proportion of statin discontinuation of statin-based therapy within patients with history of SAMS. The protocol was registered on PROSPERO (CRD42020202619).

**Results:**

In 23 studies (13 RCTs, 2 prospective and 8 retrospective studies) there were in total 1868 participants in RCTs and 47’628 participants in non-RCTs (follow-up 12 weeks – 31 months). Our confidence in the body of evidence using GRADE was moderate for the primary outcome and low-moderate for the secondary outcome. In RCTs among patients with history of SAMS, there was high heterogeneity in the statin regimens and controls (placebo/non-daily dosing/ezetimibe/PCSK9 inhibitors). In RCTs, the meta-analysis showed no difference between statin-based and control groups in the incidence of muscle symptoms (OR 1.19, 95%CI 0.86–1.64, I^2^: 46.3%)). Therapy discontinuation due to muscle symptoms in RCTs was higher in the statin-based than the comparator groups (OR 1.48, 95%CI 1.03–2.12, I^2^ = 17.6%).

**Conclusion:**

Our findings suggest that patients with a history of SAMS can be re-challenged with statins. More high-quality evidence is needed to strengthen guidelines regarding the management of SAMS.

## Introduction

Statin-associated muscle symptoms (SAMS) pose a significant clinical challenge in both primary and secondary prevention of cardiovascular events. Statins serve as the cornerstone in mitigating cardiovascular risk and reducing mortality [1,2]. They are widely prescribed with increased intensity to achieve currently recommended Low-Density Lipoprotein cholesterol (LDL-C) levels [3–5]. Nevertheless SAMS is a common adverse effect occurring in approximately 10% (95% CI: 8.1–10%) of patients taking a statin. However the prevalence of statin-associated muscle symptoms varies across populations and is influenced by factors such as female sex, older age, obesity, and statin intensity [6]. Intolerance to statins is linked with an elevated risk of cardiovascular disease [3]. Meanwhile, the non-statin alternative medications such as PCSK9 inhibitors suffer from substantial costs [7] and have been mainly studied in large RCTs as add-on treatments on top of statins [8]. Consequently, statins maintain their status as the primary treatment option [9,10].

The underlying pathophysiological mechanism of SAMS currently remains unclear [11]. One hypothesis involves mitochondrial dysfunction: statins may impair mitochondrial respiration and reduce the synthesis of coenzyme Q10 (ubiquinone), leading to decreased ATP production and increased oxidative stress in muscle cells [12]. Additionally, statins can interfere with calcium signaling and activate muscle atrophy pathways [13]. These combined effects may contribute to muscle fiber damage and the clinical manifestations of SAMS.

The 2015 European Atherosclerosis Society Consensus Panel Statement recommended multiple strategies to optimize statin tolerance in patients with previous SAMS based on expert opinion due to lack of sufficient data [14,15]. To date, the results of systematic reviews and meta-analysis are limited and focused on statin-naïve patients [16–18]. The most recent systematic reviews and meta-analyses on this topic evaluated the effectiveness of intermittent, non-daily statin administration compared with daily dosing in patients with or without a history of statin-associated muscle symptoms (SAMS). All three studies [16–18] applied differing definitions of statin-associated muscle symptoms (SAMS), contributing to variability in outcome assessment and limiting comparability across findings. The results revealed conflicting evidence regarding the incidence of muscle symptoms [16,18]. To date, no meta-analysis has exclusively focused on patients with a history of SAMS, limiting the evidence base for treatment strategies in this specific population at increased risk of statin-associated side effects. By focusing solely on patients with documented prior SAMS and examining how outcome definitions vary across studies, the present review not only addresses this evidence gap but also underscores the need for a standardized definition of SAMS to improve consistency in future research and clinical practice. Recent narrative reviews on the management of SAMS found that further research is needed to close the knowledge gap [19]. New evidence on the management of SAMS have since become available both from randomized control trials (RCTs) and from observational data [20–26]. This systematic review therefore aimed to investigate the incidence of muscle symptoms, analyze statin-therapy discontinuation rates, and assess the effectiveness of statin-based therapeutic management in individuals with a history of SAMS. Furthermore, we attempted to identify existing evidence gaps that help to guide the direction of future research.

## Methods

### Data sources, search strategy, selection criteria, data extraction

We followed the Preferred Reporting Items for Systematic Reviews and Meta-analyses (PRISMA) reporting guideline for meta-analysis. The protocol was registered on PROSPERO (CRD42020202619) and published elsewhere [27].

The search strategy we used for MEDLINE, EMBASE and Cochrane Central Register of Controlled Clinical Trials is provided in the Supplement (S1 File). We performed the search in the different databases from inception until February 2025. The search period was extended in comparison to the published protocol from April 2021 due to the COVID pandemic and further to February 2025 in order to be able to publish an uptodate literature search. Additionally, we conducted a hand search using forward and backwards citations and systematically evaluated the grey literature (Scopus, Clinicaltrials.gov and Proquest – (S1 File)) for additional potentially relevant and unpublished articles [28]. We included RCTs and non-randomized studies examining adults with a history of SAMS, comparing interventions testing statin-based strategies with a control group. The primary outcome was the incidence of muscle symptoms. We chose to include non-randomized trials due to the limited availability of data from RCTs. The inclusion of observational studies was necessary to provide a broader evidence base to enhance the comprehensiveness of our review. History of SAMS was defined according to the respective study protocol. We set no follow-up length restrictions. We excluded studies examining adults without a history of adverse effects due to statin therapy, children, adolescents and pregnant women. To avoid missing relevant data, we set no restriction on the type of comparator [27].

Three trained researchers evaluated (FV, CL, PA) independently eligibility based on titles and abstracts and then by full-text screening. Expecting poor reporting of discontinuation of statin-therapy and adverse outcomes in titles and abstracts, we included studies in the first screening mainly based on population and intervention information. Three authors (FV, CL, PA) independently performed study selection, review, and data extraction. We extracted data from selected studies using a prewritten data extraction form. The main extracted data are listed in the protocol [27]. Disagreements were resolved by through discussion with senior investigators (MB, NR) and consensus.

### Types of outcomes

In deviation from the protocol, two outcome names were revised from “tolerability” to “incidence of muscle symptoms” and from “acceptability” to “treatment discontinuation due to muscle symptoms.” This modification was made to improve clarity and ensure more precise communication of the findings. However, the definition of the outcomes remained unchanged.The primary outcome was incidence of muscle symptoms as defined by the individual studies. We used odds ratios (OR) to compare the incidence of muscle symptoms between the statin group and the control group, with an OR >1 indicating higher incidence of muscle symptoms in the statin-based treatment group.

Secondary outcomes included treatment discontinuation due to muscle symptoms, and effectiveness defined as change in LDL-cholesterol from baseline. We used ORs to compare the discontinuation rates between the statin group with the control group, with an OR >1 indicating higher discontinuation rates of the statin-based treatment group.

### Quality of evidence

Three reviewers evaluated (FV, CL, PA) independently the risk of bias of each study using the Risk-of-Bias 2 tool for RCTs, the ROBINS-I tool from the Cochrane Collaboration tools for prospective cohort studies [29] and the Newcastle-Ottawa scale for retrospective cohort studies, which was converted to per AHRQ criteria into a summary quality rating [30]. The overall quality of evidence was assessed by using GRADE (Grading of Recommendations Assessment, Development and Evaluation working group methodology) [31]. Discrepancies between reviewers were solved by consensus or by a third person.

### Statistical analysis

The statistical analyses were done using STATA software version 15 and 16 (StataCorp, College Station, Texas, USA). The visual presentation of the meta-analysis was created using Adobe Illustrator (Adobe, San Jose, California, USA)We synthesized the systematic review quantitatively and qualitatively.

For the qualitative synthesis, we summarized the characteristics and findings of the included studies in text and tables. We categorized our summaries of studies according to type of intervention, comparator, outcome, and study design. Limitations of included studies as well as recommendations for future research were synthetized. Median age was weighted by study size, as the studies vary highly in included participants.

We analyzed data from RCTs for the quantitative synthesis. Studies that did not report dichotomous data on SAMS were excluded, as it was not possible to determine an appropriate cut-off to convert continuous data into a meaningful dichotomous format. We separated trials in subgroups according to their control treatment to reduce overall heterogeneity. We pooled results using random-effects meta-analysis, expressing results as ORs with 95% confidence intervals (CI) for incidence of muscle symptoms and treatment discontinuation. If a group of trials reported zero events, we used the zero count-cell method [32]. We excluded studies with zero events in both groups from the analysis. We defined high heterogeneity as >40% [33].

For the implementation of cross-over trials in the meta-analysis, we used the method according to Becker/Balagtas [34]. This method ensures that no participants were counted double in the meta-analysis. As this method is performed using OR, we decided to use OR in our meta-analysis to ensure methodological validity. Where needed, we contacted authors to obtain exact data for within-individual comparison of treatments.

## Results

### Search results

**Fig 1:**
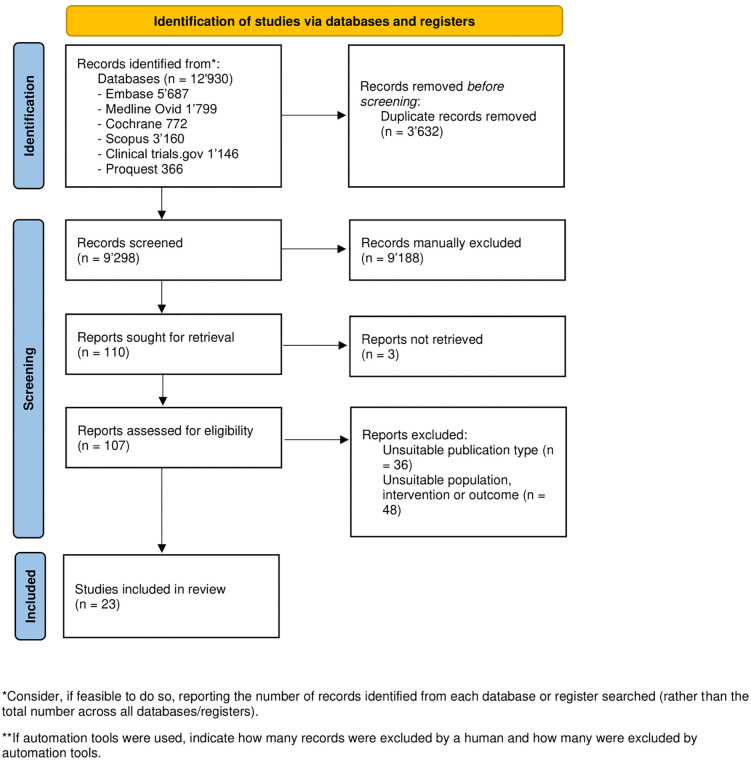
PRISMA 2020 flow diagram, n = number of studies.

The search identified 9’298 unique records. We retained 110 records for full text assessment. 23 studies were included for data extraction [20–26,35–50], with 9 RCTs analyzed quantitatively. The inter-rater reliability for the initial title and abstract screening phase in Cohen’s kappa was 0.58. No further studies were identified by hand search, however an unpublished trial was identified on clinicaltrials.gov and included in the analysis. The PRISMA flow diagram is shown in Fig 1.

### Quality of evidence

We judged 8 [20,22,23,25,26,40,48,49] out of 13 RCTs at low risk of bias (S2 File). Three trials showed some concern on the randomization process [43,46,47]. Two trials did not provide enough information on pre-specified outcomes to allow assessment of reporting bias [21,47]. Insufficient information was provided regarding the methodology to address carryover effects during the data analysis in two crossover trials [24,43].

The two prospective cohort studies were both of low quality, mainly due to concerns regarding residual confounding and ascertainment of outcomes (S2 File) [37,39]. Notably all 8 identified retrospective studies were of low quality, mainly due to concerns regarding residual confounding because of lack of adjustment (S2 File) [35,36,38,41,42,44,45,50].

We estimated our confidence in the resulting body of evidence using GRADE as moderate for the outcome of incident muscle symptoms and low to moderate for the outcome of discontinuation of statin treatment [31] (S3 File).

### Characteristics of studies of the randomized controlled trials

We included 13 RCTs conducted between 2006–2023, of which five were parallel RCTs [40,46–49], five were crossover RCTs [20,21,23,26,43] and three were n-of-1 RCTs [22,24,25] (S4 File, Table 1). The number of participants ranged from 8 to 491 with a median of 101 participants. The weighted mean age was 63.2 years. Interventions and comparators were diverse: nine trials assessed statin against placebo [20–26,43], two trials statin against PCSK9 inhibitors [46,47], one trial statin against red yeast rice [40], one trial daily versus non-daily statin dosing and two trials statin with ezetimibe against ezetimibe alone [46,48]. In all trials, with the exception of one, both patients and investigators were blinded to the treatment allocation. Most trials used statins with low to moderate intensity [20,21,24–26,40,43,46,48,49], two trials used high-intensity statins [23,47]. The exposure time ranged from 12 weeks to 12 months with a weighted mean exposure time of 22 weeks.

Reporting of outcomes of interest was diverse. Ten trials [20,21,25,26,40,43,46–49] provided data on incidence of muscle symptoms, whereof most trials [22,23,25,40,49] used a mean difference in pain intensity between statin and comparator group, and two trials [24,26] used symptom intensity expressed as a nocebo ratio. Nine trials [22,25,26,40,43,46–48] provided data for treatment discontinuation due to muscle symptoms, and seven trials [40,43,46–49] reported data on effectiveness.

The trials comparing statin-based therapy to placebo showed similar between-group incidence of muscle symptoms and statin-discontinuation rate. [20,21,25,26,43,47] Comparing statin-based therapy to ezetimibe only, two trials found no significant difference in muscle symptoms, but significantly lower LDL-C levels in the statin-based group [46,48]. One trial compared a statin-based therapy with red-yeast rice and found no difference in muscle symptoms or therapy discontinuation. However, the effectiveness of red-yeast rice in LDL-C reduction was similar compared to pravastatin [40]. When comparing statin-based therapy to PCSK9 inhibitors, neither of the two studies found a difference in discontinuation rate or incidence of muscle symptoms [46,47]. The only trial comparing non-daily statin dosing to daily dosing found patterns of lower muscle symptoms incidence in daily dosing but with large confidence intervals and did not report on discontinuation rate (OR 0.58 (0.23–1.43)).

### Quantitative synthesis of the randomized controlled trials

We performed meta-analysis comparing statin therapy or higher intensity statin therapy versus any alternative lipid-lowering medication or placebo in all RCTs. As the non-randomized trials were of low quality, we did not include them in the meta-analysis. Due to high heterogeneity in comparators, we split comparators into subgroups. The n-of-1 trials and a crossover-trial [23] were excluded from the meta-analysis because they reported muscle symptoms as a continuous variable, while the meta-analysis required dichotomous variables for comparability.

### Incidence of muscle symptoms (Fig 2)

Data on incidence of muscle symptoms were available in five parallel-group RCTs [40,46–49], and three crossover RCTs [20,21,43]. The analysis showed high heterogeneity (overall I^2^: 46.3%); the odds ratio (OR) was 0.96 (95% CI 0.53–1.73) for statin vs. ezetimibe, and 1.57 (95%CI 0.92–2.66) for statin vs PCSK9 inhibitors, and 1.27 (95% CI 0.77–2.08) for statin vs. placebo. Non-daily vs. daily statin dosing resulted in an OR of 0.58 (95% CI 0.23–1.43). [34,51,52]. Overall, the OR for the incidence of muscle symptoms was 1.19 (0.86–1.64), however there was high methodological and statistical heterogeneity in the trials.

**Fig 2:**
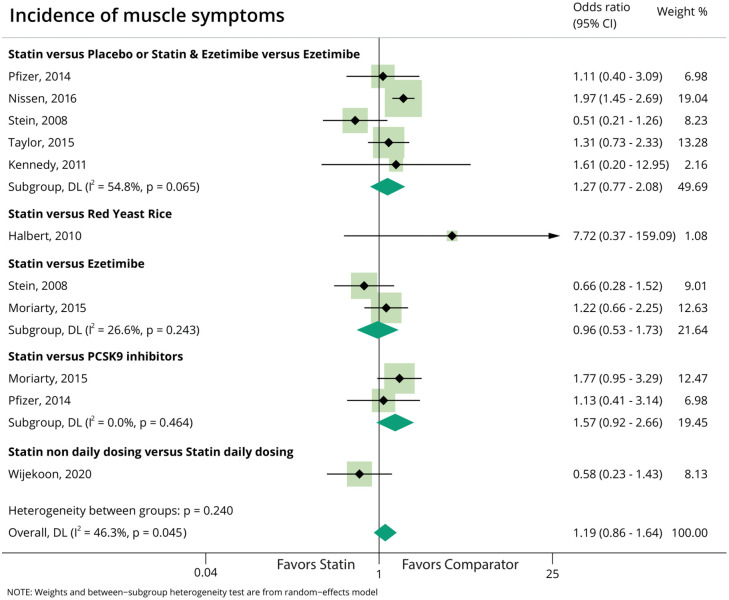
Meta-analysis of incidence of muscle symptoms during intervention phase, comparing due to statin-based therapy vs. different various comparators.

### Treatment discontinuation due to muscle symptoms (Fig 3)

**Fig 3:**
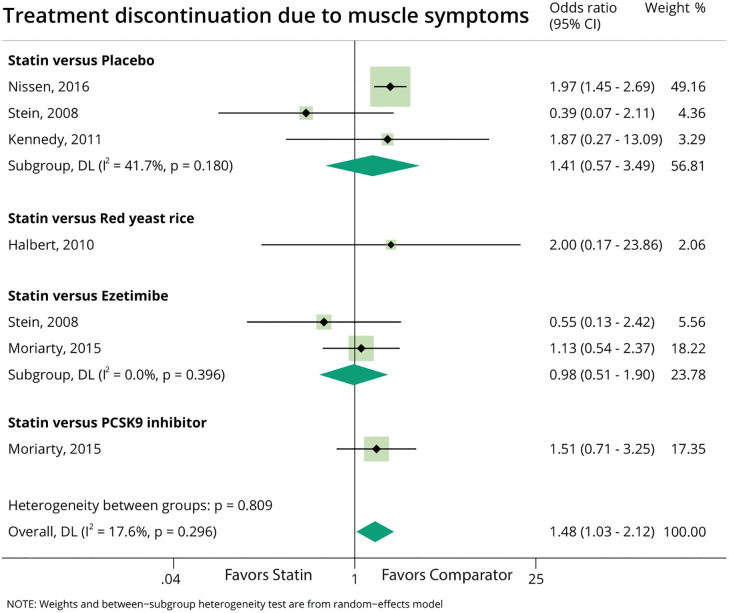
Meta-analysis of treatment discontinuation due to muscle symptoms during intervention phase, comparing due to statin-based therapy vs. different various comparators.

Data on treatment discontinuation were available in three parallel-group RCTs [40,46,48], and two crossover RCTs [20,43]. Overall, the OR for treatment discontinuation due to muscle symptoms was 1.48 (95% CI 1.03–2.12), indicating higher treatment discontinuation in the statin-based treatment strategies (Fig 3). In the subgroup analysis the OR was 0.98 (95%CI 0.51–1.90) for statin vs. ezetimibe, and 1.41 (95%CI 0.57–3.49) for statin vs. placebo. Statistical heterogeneity was low (I^2^ = 17.6%).

### Effectiveness

We were not able to pool the data for effectiveness, as only three parallel-group RCTs [40,47,49] reported effectiveness with standard deviations, with each trial using a different comparator.

A leave-one-out analysis and a subgroup analysis stratified by statin dosage to determine the cause of high heterogeneity was conducted and can be found in Supporting information S5–S7 File. It was not possible to perform the preplanned subgroup analyses on cardiovascular prevention setting, statin dosing strategy, level of intolerance, co-interventions and presence of statin therapy at inclusion, due to high diversity in study designs and comparators.

### Characteristics of studies of the non-randomized trials

We included eight retrospective cohort studies [35,38,39,41,42,44,45,50] and two cohort prospective studies [36,37] (S4 File, Table 2). The weighted median age was 62.9 years and mean follow-up duration was 14.5 months. The number of participants ranged from 27 to 45’037 with a median of 113 participants.

Exposures and comparators were highly variable, with different non-statin lowering agents as comparators and various dosing strategies. Reporting of outcomes of interest was diverse. Three studies reported the incidence of muscle symptoms [36–38], five studies provided data on treatment discontinuation due to muscle symptoms [36,37,39,42,45], and eight studies reported on effectiveness [35–37,39,41,44,45,50].

The first prospective cohort study compared 5 mg rosuvastatin to 10 mg rosuvastatin and found similar muscle symptoms, discontinuation rate and effectiveness [39].

The other prospective cohort study compared statin-based therapy combined with a herbal tincture (*Berberis aristata* and *Silybum marianum*) to ezetimibe and the herbal tincture, and found no significant difference in incidence of muscle symptoms [37]. The addition of various co-interventions was also analyzed by several retrospective studies. The addition of phytosterols + psyllium, the previously mentioned herbal tincture or fenofibrate to ezetemibe was not associated with a change of muscle symptoms; however, the addition of fenofibrate to ezetemib correlated with a higher discontinuation rate. On the other hand, a different study reported lower incidence of muscle symptoms was reported when comparing statin-based treatment with fibrates, bile sequestrant or omega-3 fatty acids [38]. A small retrospective cohort study found non-statin lipid-lowering monotherapy (including ezetimibe, fibrate, bile acid sequestrant, or omega-3 fatty acids) to be ineffective in patients with statin intolerance in comparison to statin-based treatments.

Retrospective studies also investigated different intensities and frequencies of statin therapy. A retrospective cohort study comparing the incidence of muscle symptoms of daily, bi-weekly, and weekly rosuvastatin therapy showed that the reduction of LDL-C levels with once-weekly rosuvastatin therapy was still 20%, compared to reduction of 40% with a daily dosage [45]. In comparison, the reduction in LDL-C levels was also around 20% with intermittent dosing of statin-therapy in a different study [44]. When comparing effectiveness and discontinuation rates of different statin-based therapy intensities found that the discontinuation rate was similar across intensities [41,42]. This was also reported when comparing a same-statin rechallenge, statin switch and a non-statin based therapy [35].

### Publication bias

The high methodological and statistical heterogeneity of interventions in our meta-analysis precluded the assessment of publication bias with statistical methods, e.g., using funnel plots or the Egger Test. When interventions vary greatly, as in our case, the resulting differences in effect sizes and outcomes introduce substantial variability that can obscure the patterns these methods aim to detect [53].

Despite these limitations, our search in grey literature uncovered an unpublished trial. The existence of this unpublished study suggests that publication bias may be present, as it indicates a potential discrepancy between published and unpublished research.

## Discussion

In this systematic review and meta-analysis of 23 studies, the incidence of muscle symptoms appeared similar between groups (OR 1.19); however, the wide confidence interval (0.86–1.64) includes both the possibility of harm and no effect and thus should be interpreted with caution. More patients discontinued statin therapies over the trial duration compared to non-statin therapies (OR 1.48, 95% CI 1.03–2.12).The higher discontinuation rate observed with statins may reflect challenges in patient adherence and could have implications for long-term cardiovascular risk prevention [3]. High-quality evidence such as large RCTs are missing. As SAMS remain a major obstacle in lipid-lowering treatment, this review helps to strengthen recommendations on the management of SAMS in clinical practice and guide future research. A recent systematic review and meta-analysis evaluated intermittent non-daily versus daily statin administration and included a broad range of study designs—three case reports, five retrospective cohort studies, one prospective cohort, and one randomized trial. Importantly, this review did not focus exclusively on patients with prior statin-associated muscle symptoms (SAMS), limiting the applicability of its findings to this high-risk population. Additionaly intermittent dosing is only one possible approach to manage SAMS. In 14, our analysis specifically targets patients with documented SAMS allowing all clinically relevant evaluation of treatment strategies in this population [16].

The present review extends these findings through including a broader array of studies, including 13 RCTs, two retrospective studies, and eight prospective studies. In addition to assessing the incidence of muscle symptoms, we also considered the discontinuation rate and effectiveness, although the data on effectiveness were too diverse to draw conclusive results. Analyzing the discontinuation rate provides insights into the real-world effectiveness of the therapy, thereby informing daily clinical practice.

The present analysis also includes N-of-1 trials that were proposed in recent research to optimize personalized treatments. They produce intrapersonal evidence gaining perspective into an individual patient’s care. The multiple exposures help to control for confounders as muscle symptoms are common and could occur incidentally and not be associated with statin. Therefore, n-of-1 trials can help to identify a possible nocebo effect under statin treatment. On the contrary, n-of-1 trials demand long-term investment of each individual, which can lead to high rates of missing data [22,25].

A challenge was the inconsistent definition of SAMS in inclusion criteria as well as in the outcome measurements. The studies varied in minimum number of previously tried statin [35,39,40,46] or various creatinine kinase thresholds [37,43]. Furthermore, the timing of the measurement of muscle symptoms is important. SAMS usually occur 4–6 weeks after statin introduction and can last for up weeks to months after statin therapy discontinuation [14,54]. Although many studies implemented wash-out periods [23] or a threshold time for SAMS occurrence [22,49], this presents a difficulty for cross-over designs and particularly n-of-1 trials. In earlier studies, SAMS were assessed through incidence of SAMS [17,43,46–48] and later quantified with the VAS (visual analogue scale) [22,23,25,40,49]. With suggestion of a nocebo effect, trials started reporting the nocebo ratio, e.g., the symptom intensity in placebo periods in relation to the symptom intensity when taking a statin [24,26]. There is a need for a standardized assessment of definition SAMS and outcomes assessment. Several non-invasive diagnostic tools have been evaluated [55,56], we propose the use of the SAMS-CI Score [57] due to its feasibility for both clinical and research purposes.

### Strengths and limitations

Given the significant variability in current clinical practices regarding the management of SAMS, the strength of our meta-analysis lies in the broad selection criteria used for the included studies. This variability is evident in the current guidelines, which mostly rely on expert opinions. [6,9]. In addition, we applied broad selection criteria during the screening process to include studies for full-text screening, even if they provided limited reporting of adverse events in the title or abstract. Due to the limited availability of data from RCTs, non-randomized trials were included in the systematic review. The inclusion of observational studies was necessary to enhance the comprehensiveness of our review. The findings from non-randomized studies were, however, generally of low quality. Thus, they should be interpreted with caution and in the context of their methodological limitations. Through this thorough literature search, we were able to also include data from unpublished trials.

This variability is reflected in the studies we analyzed, which utilized different definitions for SAMS, different comparators ranging from placebo to phytotherapeutics and newer lipid-lowering therapies such as PCSK-9 inhibitors [20,40,46]. This variability is also reflected in the statistical heterogeneity observed in the meta-analysis. This is due to the wide variety of study designs, the wide confidence intervals of individual studies, and the inclusion of low-weight studies, which can have a disproportionate effect on heterogeneity, especially in random-effects models [58]. Subgroup analysis showed that the heterogeneity stems mainly from the analysis of the “Statin versus Placebo or Statin & Ezetimibe versus Ezetimibe” subgroup, which exhibited the highest within-group heterogeneity (I² = 54.8%). Within this subgroup, the study by *Nissen et al.* was the principal contributor to heterogeneity, given its relatively strong effect size (OR = 1.97) and large statistical weight (19.0%). Sensitivity analysis excluding *Nissen et al.* led to a marked drop in overall heterogeneity and a corresponding narrowing of confidence intervals, without a meaningful shift in the direction of the effect estimate. (S5 File) A leave-one-out sensitivity analysis did not identify additional major contributors to heterogeneity. When excluding the *Statin versus Placebo or Statin & Ezetimibe versus Ezetimibe* arm of the trial by Stein et al. [48], the overall pooled effect size increased from OR 1.19 to OR 1.74 (S6 File). This shift likely reflects the fact that Stein et al. was the only study investigating a moderate- to high-intensity statin. Consistently, a stratified meta-analysis by statin intensity demonstrated a reduction in heterogeneity when restricted to low-intensity statin trials (S7 File). Although the pooled OR also differed between the intensity, this difference should be interpreted cautiously given that the moderate- to high-intensity subgroup comprised only the single study by Stein et al. Overall, these findings suggest that the observed heterogeneity was mainly driven by two factors: the highly weighted study by Nissen et al. [20] and the inclusion of a higher intensity statin in the Stein et al. [48] trial. This suggests that the heterogeneity was largely driven by this single influential study rather than systematic differences across all included studies. Therefore, creating a universal recommendation for clinicians remains challenging, underscoring the importance of individualized patient assessment and management.

Our analysis used odds ratios in the meta-analysis. OR have the tendency of overestimating effects especially in common events. In order to include cross-over trials in our trials using the method by Becker/Balagtas, OR was used to provide a stable effects measure [34].

As the trials included in our systematic review focus on patients with a previous history of SAMS, the population may not represent the general population with SAMS in a clinical setting. Our GRADE assessment indicates that the overall certainty of evidence was moderate for incident muscle symptoms and low to moderate for discontinuation of statin treatment. This reflects limitations in trial methodology, heterogeneity across outcome definitions, and concerns regarding residual confounding in observational studies. As a result, while our findings provide important insights, the resulting recommendation from the evidence is weak for both outcomes. The quality of non-randomized study was generally poor and the outcomes of interest of our systematic review were not the main outcomes or the pre-specified outcomes of some studies [20,46,47]. We did not have a pre-specified plan in the protocol for dealing with non-randomized trials, but we ultimately decided not to include them in the quantitative analysis due to their low quality.

### Clinical and research impact

This review suggests that statin re-challenge may be feasible in patients experiencing statin-associated muscle symptoms (SAMS), as the incidence of muscle-related symptoms and treatment discontinuation appears comparable between statin-based therapies and alternative treatment options. If future research confirms the higher discontinuation rate observed with statins in our analysis, this would underscore potential challenges in long-term patient adherence, which may in turn affect the durability of LDL-C reduction and overall cardiovascular risk prevention. Future research should include blinded placebo controlled studies with a long enough washout period after statin therapy and an agreed-upon definition of SAMS (e.g., SAMS-CI Score) at inclusion and as an outcome measurement. This will decrease methodological heterogeneity, thereby improving future meta-analyses. In our review, we used the original main outcome definitions as reported in each study without further harmonization across trials. Establishing a consistent definition would have required access to individual participant data, which was beyond the scope of our analysis. If feasible, future trials should aim to obtain individual participant data (IPD) to enable standardized definitions of SAMS and consistent outcome measurement. Trials should consistently report co-interventions such as diet changes or increased physical exercise.

In addition, trials should assess LDL-C change from baseline and hard cardiovascular outcomes for evaluation of cardiovascular prevention benefit of different tolerated lipid-lowering strategies. Finally, further studies should also focus not only on intensity of muscle pain but also on the impact of the muscle symptom on general quality of life, falls and hospitalizations.

## Conclusion

We found similar incidence of muscle symptoms between the statin-based management and the various comparator groups in patients with a previous history of SAMS. In comparison to the incidence of muscle symptoms, the discontinuation proportions were low. This suggests that in patients with prior SAMS, a re-challenge with statin therapy should be aimed for in clinical practice, implemented alongside shared decision-making and close clinical monitoring, to achieve LDL-C reduction and cardiovascular risk prevention.

## Supporting information

S1 File  Full search strategy per database. (DOCX)

S2 File Risk of bias assessments. (DOCX)

S3 File GRADE table for included RCTs. (DOCX)

S4 File Table of included studies. (DOCX)

S5 File Sensitivity analysis of the incidence of SAMS by removing Nissen et al. (DOCX)

S6 File Leave-on-out analysis on the incidence of muscle symptoms. (DOCX)

S7 File Stratified analysis by statin-intensity. (DOCX)

S8 File List of included and excluded studies. (DOCX)

S9 File Extracted data sheet. (XLSX)

S10 File Prisma checklist. (DOCX)
