# Supplementary material for: Safety outcomes of statin vs non-statin lipid-lowering interventions in patients with prior statin-associated muscle symptoms: A systematic review and meta-analysis
Source: PLoS One. 2025 Dec 11;20(12):e0338575. doi: 10.1371/journal.pone.0338575 (PMC12698018; doi:10.1371/journal.pone.0338575)
Supplement: S1 File — (DOCX) [file pone.0338575.s001.docx]

S2 Supporting information 2: Full Search Strategy per Database

Embase.com

| # | Searches | Records |  |
| --- | --- | --- | --- |
| #1 | 'antilipemic agent'/exp | 411224 | STATINS |
| #2 | 'anticholesteremic':ab,kw,ti,tn OR 'antihypercholesteremic':ab,kw,ti,tn OR 'altocor':ab,kw,ti,tn OR 'atorvastatin':ab,kw,ti,tn OR 'baycol':ab,kw,ti,tn OR 'bervastatin':ab,kw,ti,tn OR 'canef':ab,kw,ti,tn OR 'cerivastatin':ab,kw,ti,tn OR 'compactin':ab,kw,ti,tn OR 'cranoc':ab,kw,ti,tn OR 'crestor':ab,kw,ti,tn OR 'crilvastatin':ab,kw,ti,tn OR 'dalvastatin':ab,kw,ti,tn OR 'fluindostatin':ab,kw,ti,tn OR 'fluvastatin':ab,kw,ti,tn OR 'glenvastatin':ab,kw,ti,tn OR 'hmg coa*':ab,kw,ti,tn OR 'hydroxymethylglutaryl*':ab,kw,ti,tn OR 'hypochol*':ab,kw,ti,tn OR 'hypolipidemic':ab,kw,ti,tn OR 'lescol':ab,kw,ti,tn OR 'lipitor':ab,kw,ti,tn OR 'lipex':ab,kw,ti,tn OR 'lipostat':ab,kw,ti,tn OR 'livalo':ab,kw,ti,tn OR 'locol':ab,kw,ti,tn OR 'lovostatin':ab,kw,ti,tn OR 'lochol':ab,kw,ti,tn OR 'medostatin':ab,kw,ti,tn OR 'mevacor':ab,kw,ti,tn OR 'mevalotin':ab,kw,ti,tn OR 'mevinacor':ab,kw,ti,tn OR 'mevinolin':ab,kw,ti,tn OR 'monacolin':ab,kw,ti,tn OR 'pitava':ab,kw,ti,tn OR 'pitavastatin':ab,kw,ti,tn OR 'pravachol':ab,kw,ti,tn OR 'pravasin':ab,kw,ti,tn OR 'pravastatin':ab,kw,ti,tn OR 'rosuvastatin':ab,kw,ti,tn OR 'simvastatin':ab,kw,ti,tn OR 'zocor':ab,kw,ti,tn OR 'statin*':ab,kw,ti,tn | 141773 |  |
| #3 | #1 OR #2 | 443854 |  |
| #4 | 'muscle disease'/exp | 623678 | SAMS |
| #5 | (((muscl* OR muscu*) NEAR/3 (pain* OR fatigue OR intoler* OR symptom*)):ti,ab,kw) OR ((statin* NEAR/3 (induc* OR relat* OR associ* OR intoler* OR symptom* OR 'side effect*' OR side-effect* OR 'adverse effect*')):ti,ab,kw) OR sams:ti,ab,kw OR sas:ti,ab,kw OR sinam:ti,ab,kw OR myopath*:ti,ab,kw OR myalg*:ti,ab,kw OR myosit*:ti,ab,kw OR necrot*:ti,ab,kw OR rhabdomyo*:ti,ab,kw | 411088 |  |
| #6 | #4 OR #5 | 924157 |  |
| #7 | #3 AND #6 | 35804 |  |
| #8 | 'dosage schedule comparison'/exp OR 'drug choice'/exp OR 'drug dose comparison'/exp OR 'drug dose escalation'/exp OR 'drug dose increase'/exp OR 'drug dose intensification'/exp OR 'drug dose reduction'/exp OR 'drug dose regimen'/exp OR 'drug dose titration'/exp OR 'drug intermittent therapy'/exp OR 'drug megadose'/exp OR 'drug microdose'/exp OR 'drug pulse therapy'/exp OR 'low drug dose'/exp OR 'loading drug dose'/exp OR 'maintenance drug dose'/exp OR 'recommended drug dose'/exp OR 'optimal drug dose'/exp | 654428 | MANAGEMENTS |
| #9 | (((daily OR everyday OR quotidian OR 'every other' OR 'every second' OR rotat* OR alternat* OR 'non daily' OR nondaily OR non-daily OR hebdomad* OR weekly OR intermit* OR escalat* OR build-up OR intensif* OR reduct* OR puls*) NEAR/3 (therap* OR dos* OR treatment OR prescript*)):ti,ab,kw) OR reinitiation:ti,ab,kw OR challeng*:ti,ab,kw OR rechalleng*:ti,ab,kw OR 're challeng*':ti,ab,kw or monotherap*:ti,ab,kw | 2318527 |  |
| #10 | #8 OR #9 | 2850203 |  |
| #11 | #7 AND #10 | 7975 |  |
| #12 | 'crossover procedure':de OR 'double-blind procedure':de OR 'randomized controlled trial':de OR 'single-blind procedure':de OR random*:de,ab,ti OR factorial*:de,ab,ti OR crossover*:de,ab,ti OR ((cross NEXT/1 over*):de,ab,ti) OR placebo*:de,ab,ti OR ((doubl* NEAR/1 blind*):de,ab,ti) OR ((singl* NEAR/1 blind*):de,ab,ti) OR assign*:de,ab,ti OR allocat*:de,ab,ti OR volunteer*:de,ab,ti | 3483850 | RCT |
| #13 | cohort:ab,ti OR (case:ab,ti AND (control:ab,ti OR controll*:ab,ti OR comparison:ab,ti OR referent:ab,ti)) OR risk:ab,ti OR causation:ab,ti OR causal:ab,ti OR 'odds ratio':ab,ti OR etiol*:ab,ti OR aetiol*:ab,ti OR 'natural history':ab,ti OR predict*:ab,ti OR prognos*:ab,ti OR outcome:ab,ti OR course:ab,ti OR retrospect*:ab,ti OR 'epidemiology'/de | 11447054 | OBSSTU |
| #14 | #12 OR #13 | 13500435 |  |
| #15 | #11 AND #14 | 5,687 |  |

OVID mEDLINE

| # | Searches | Records |  |
| --- | --- | --- | --- |
| 1 | exp hypolipidemic Agents/ | 171114 | STATINS |
| 2 | (Anticholesteremic or antihypercholesteremic or altocor or atorvastatin or baycol or bervastatin or canef or cerivastatin or compactin or cranoc or crestor or crilvastatin or dalvastatin or fluindostatin or fluvastatin or glenvastatin or hmg coa* or hydroxymethylglutaryl* or hypochol* or hypolipidemic* or lescol or lipitor or lipex or lipostat or livalo or locol or lovostatin or lochol or medostatin or mevacor or mevalotin or mevinacor or mevinolin or monacolin or pitava or pitavastatin or pravachol or pravasin or pravastatin or rosuvastatin or simvastatin or zocor or statin*).ab,kw,ti,nm. | 111326 |  |
| 3 | 1 or 2 | 211485 |  |
| 4 | exp muscular diseases/ | 204358 | SAMS |
| 5 | (((muscl* or muscu*) adj3 (pain* or fatigue or intoler* or symptom*)) or (statin* adj3 (induc* or relat* or associ* or intoler* or symptom* or side effect* or side-effect* or adverse effect*)) or sams or sas or sinam or myopath* or myalg* or myosit* or necrot* or rhabdomyo*).ti,ab,kw. | 240293 |  |
| 6 | 4 or 5 | 399997 |  |
| 7 | 3 and 6 | 12567 |  |
| 8 | exp drug therapy/ | 1549622 | MANAGEMENT |
| 9 | (((daily or everyday or quotidian or every other or every second or rotat* or alternat* or non daily or nondaily or non-daily or hebdomad* or weekly or intermit* or escalat* or build-up or intensif* or reduct* or puls*) adj3 (therap* or dos* or treatment or prescript*)) or reinitiation or challeng* or rechalleng* or re challeng* or monotherap*).ab,kw,ti. | 1770929 |  |
| 10 | 8 or 9 | 3169420 |  |
| 11 | 7 and 10 | 2431 |  |
| 12 | Randomized controlled trial.pt. | 631786 | RCT |
| 13 | controlled clinical trial.pt. | 95678 |  |
| 14 | Randomi?ed.ab. | 810685 |  |
| 15 | placebo.ab. | 255981 |  |
| 16 | drug therapy.fs. | 2781468 |  |
| 17 | randomly.ab. | 453088 |  |
| 18 | trial.ab. | 738022 |  |
| 19 | groups.ab. | 2807759 |  |
| 20 | or/12-19 | 6227309 |  |
| 21 | exp animals/ not humans.sh. | 5307032 |  |
| 22 | 20 not 21 | 5458238 |  |
| 23 | Epidemiologic studies/ | 9659 | OBSERVATION STUDY |
| 24 | exp case control studies/ | 2707799 |  |
| 25 | exp cohort studies/ | 2506754 |  |
| 26 | Case control.tw. | 169245 |  |
| 27 | (cohort adj (study or studies)).tw. | 383989 |  |
| 28 | Cohort analy$.tw. | 14332 |  |
| 29 | (Follow up adj (study or studies)).tw. | 59760 |  |
| 30 | (observational adj (study or studies)).tw. | 194135 |  |
| 31 | Longitudinal.tw. | 367103 |  |
| 32 | Retrospective.tw. | 876260 |  |
| 33 | Cross sectional.tw. | 609359 |  |
| 34 | Cross-sectional studies/ | 532378 |  |
| 35 | or/23-34 | 4197480 |  |
| 36 | 22 or 35 | 8341159 |  |
| 37 | 11 and 36 | 1799 |  |

Cochrane Library

| # | Searches | Records |  |
| --- | --- | --- | --- |
| #1 | (Anticholesteremic or antihypercholesteremic or altocor or atorvastatin or baycol or bervastatin or canef or cerivastatin or compactin or cranoc or crestor or crilvastatin or dalvastatin or fluindostatin or fluvastatin or glenvastatin or hmg coa* or hydroxymethylglutaryl* or hypochol* or hypolipidemic* or lescol or lipitor or lipex or lipostat or livalo or locol or lovostatin or lochol or medostatin or mevacor or mevalotin or mevinacor or mevinolin or monacolin or pitava or pitavastatin or pravachol or pravasin or pravastatin or rosuvastatin or simvastatin or zocor or statin*):ti,ab, kw | 17608 | STATIN |
| #2 | (((muscl* OR muscu*) NEAR/3 (pain* OR fatigue OR intoler* OR symptom*)) OR ((statin* NEAR/3 (induc* OR relat* OR associ* OR intoler* OR symptom* OR “side effect“ OR side-effect* OR “adverse effect“)) OR sams OR sas OR sinam OR myopath* OR myalg* OR myosit* OR necrot* OR rhabdomyo*)):ti,ab,kw | 33519 | SAMs |
| #3 | #1 AND #2 | 2266 |  |
| #4 | (((daily or everyday or quotidian or “every other“ or “every second“ or rotat* or alternat* or “non daily“ or nondaily or non-daily or hebdomad* or weekly or intermit* or escalat* or build-up or intensif* or reduct* or puls*) near/3 (therap* or dos* or treatment or prescript*)) or reinitiation or challeng* or rechalleng* or “re challeng*“ or monotherap*).ab,kw,ti. | 35988 | MANAGEMENT |
| #5 | #1 AND #2 AND #4 | 772 |  |

GREY LITERATURE DATABASE AND SEARCH ENGINES

www.scopus.com

| # | Searches | Records |  |
| --- | --- | --- | --- |
|  | TITLE-ABS-KEY ( anticholesteremic OR antihypercholesteremic OR altocor OR atorvastatin OR baycol OR bervastatin OR canef OR cerivastatin OR compactin OR cranoc OR crestor OR crilvastatin OR dalvastatin OR fluindostatin OR fluvastatin OR glenvastatin OR "hmg coa*" OR hydroxymethylglutaryl* OR hypochol* OR hypolipidemic* OR lescol OR lipitor OR lipex OR lipostat OR livalo OR locol OR lovostatin OR lochol OR medostatin OR mevacor OR mevalotin OR mevinacor OR mevinolin OR monacolin OR pitava OR pitavastatin OR pravachol OR pravasin OR pravastatin OR rosuvastatin OR simvastatin OR zocor OR statin* ) AND TITLE-ABS-KEY ( ( ( muscl* OR muscu* ) W/3 ( pain* OR fatigue OR intoler* OR symptom* ) ) OR ( ( statin* W/3 ( induc* OR relat* OR associ* OR intoler* OR symptom* OR "side effect*" OR side-effect* OR "adverse effect*" ) ) OR sams OR sas OR sinam OR myopath* OR myalg* OR myosit* OR necrot* OR rhabdomyo* ) ) AND TITLE-ABS-KEY ( ( ( daily OR everyday OR quotidian OR "every other" OR "every second" OR rotat* OR alternat* OR "non daily" OR nondaily OR non-daily OR hebdomad* OR weekly OR intermit* OR escalat* OR build-up OR intensif* OR reduct* OR puls* ) W/3 ( therap* OR dos* OR treatment OR prescript* ) ) OR reinitiation OR challeng* OR rechalleng* OR "re challeng*" OR monotherap* ) | 3’039 |  |

Clinical trials

www.clinicaltrials.gov

| # | Searches | Records |  |
| --- | --- | --- | --- |
|  | (atorvastatin OR fluvastatin OR pitavastatin OR pravastatin OR rosuvastatin OR simvastatin OR statin) AND (pain OR fatigue OR intolerance OR symptom OR side OR adverse OR myopathy OR myalgia OR myositis OR rhabdomyolysis OR muscular OR muscle) | 1’077 |  |

Conference papers

www.proquest.com

| # | Searches | Records |  |
| --- | --- | --- | --- |
|  | AB,TI,IF(anticholesteremic OR antihypercholesteremic OR altocor OR atorvastatin OR baycol OR bervastatin OR canef OR cerivastatin OR compactin OR cranoc OR crestor OR crilvastatin OR dalvastatin OR fluindostatin OR fluvastatin OR glenvastatin OR “hmg coa*” OR hydroxymethylglutaryl* OR hypochol OR hypolipidemic OR lescol OR lipitor OR lipex OR lipostat OR livalo OR locol OR lovostatin OR lochol OR medostatin OR mevacor OR mevalotin OR mevinacor OR mevinolin OR monacolin OR pitava OR pitavastatin OR pivastatin OR pravachol OR pravasin OR pravastatin OR rosuvastatin OR simvastatin OR zocor OR statin*) AND AB,TI,IF(muscl* OR muscul* OR pain* OR fatigue OR intoler* OR symptom* OR SAMS OR SAS OR SINAM OR rhabdomyo* OR myosit* OR myopath* OR myalg* OR necrot*) | 349 |  |
