# Supplementary material for: Safety outcomes of statin vs non-statin lipid-lowering interventions in patients with prior statin-associated muscle symptoms: A systematic review and meta-analysis
Source: PLoS One. 2025 Dec 11;20(12):e0338575. doi: 10.1371/journal.pone.0338575 (PMC12698018; doi:10.1371/journal.pone.0338575)
Supplement: S2 File — (DOCX) [file pone.0338575.s002.docx]

Risk of bias of randomized controlled trial with RoB 2.0

The assessment was identical for both outcomes: "incidence of muscle symptoms" "treatment discontinuation due to muscle symptoms".

| **Parallel randomized controlled trials** | | | | | | | | | |
| --- | --- | --- | --- | --- | --- | --- | --- | --- | --- |
| **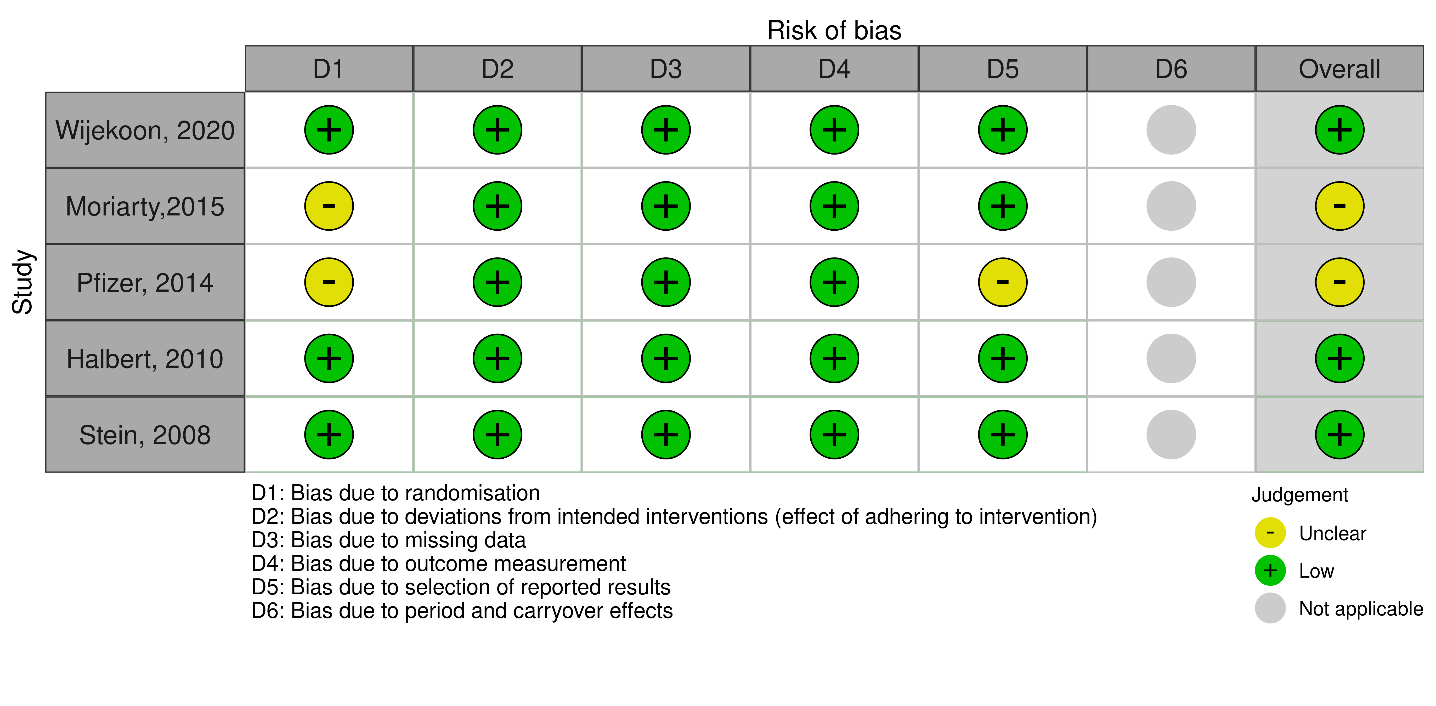**  D6: Bias due to period and carryover effects not applicable in parallel randomized controlled trials | | | | | | | | | |
| **Cross-over randomized controlled trials** | | | | | | | | | |
| 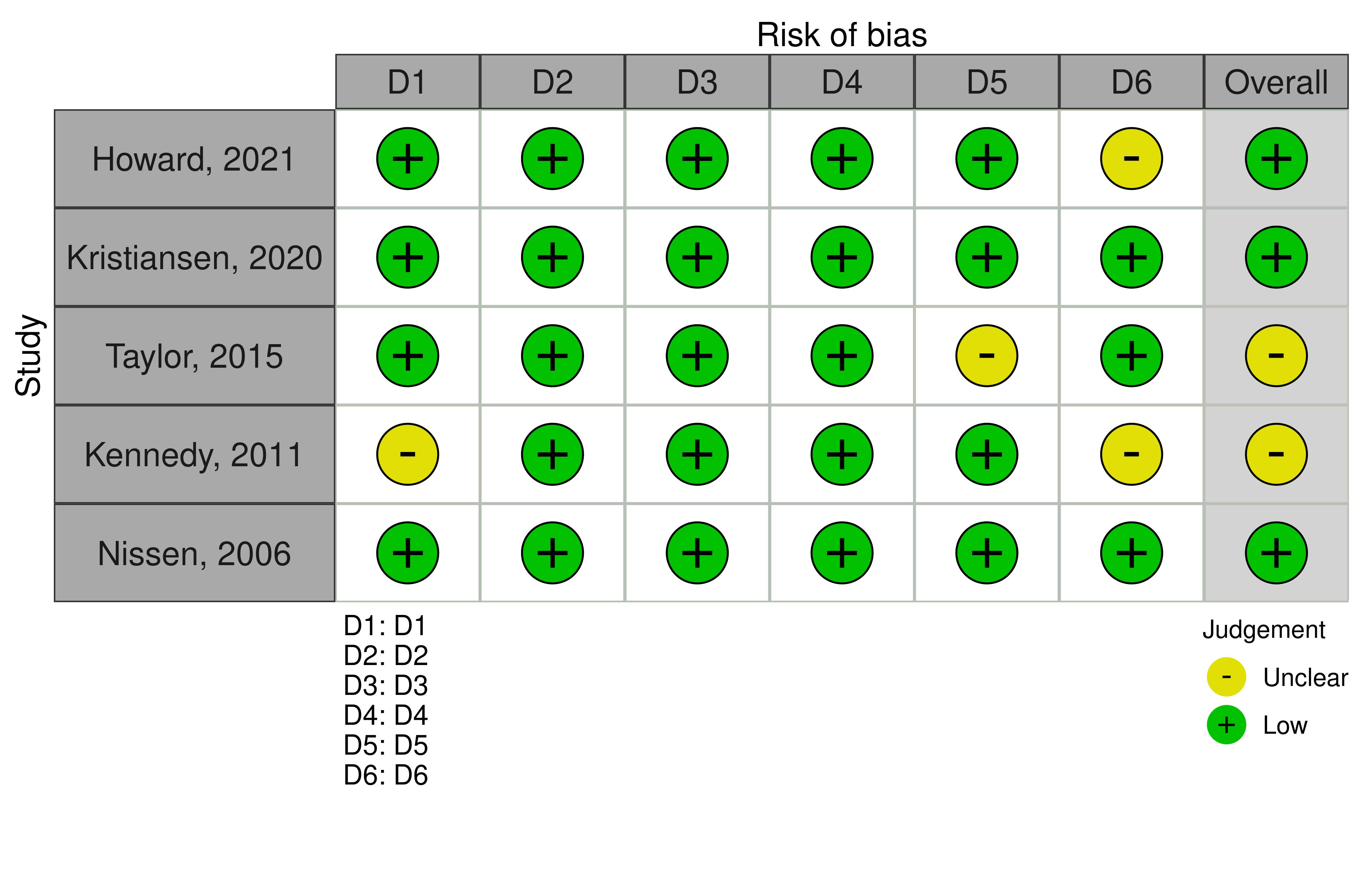 | | | | | | | | | |
| **N-of-1 Randomized controlled trials** | | | | | | | | | |
| 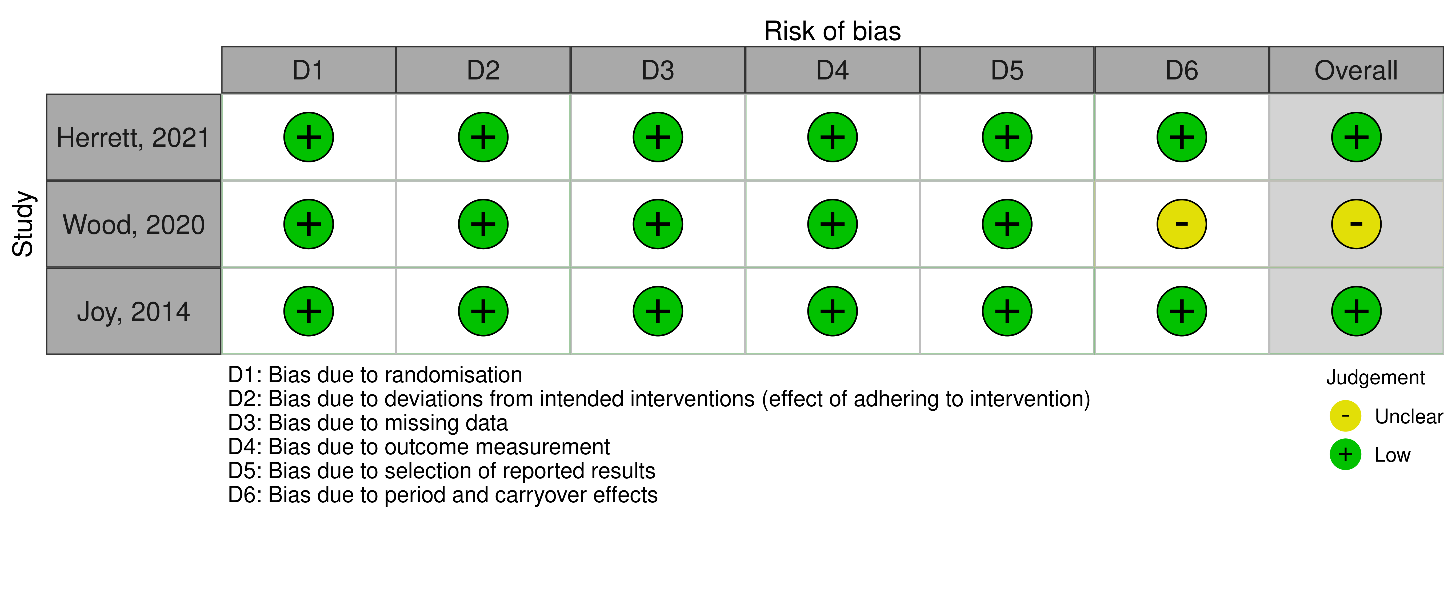 | | | | | | | | | |
| 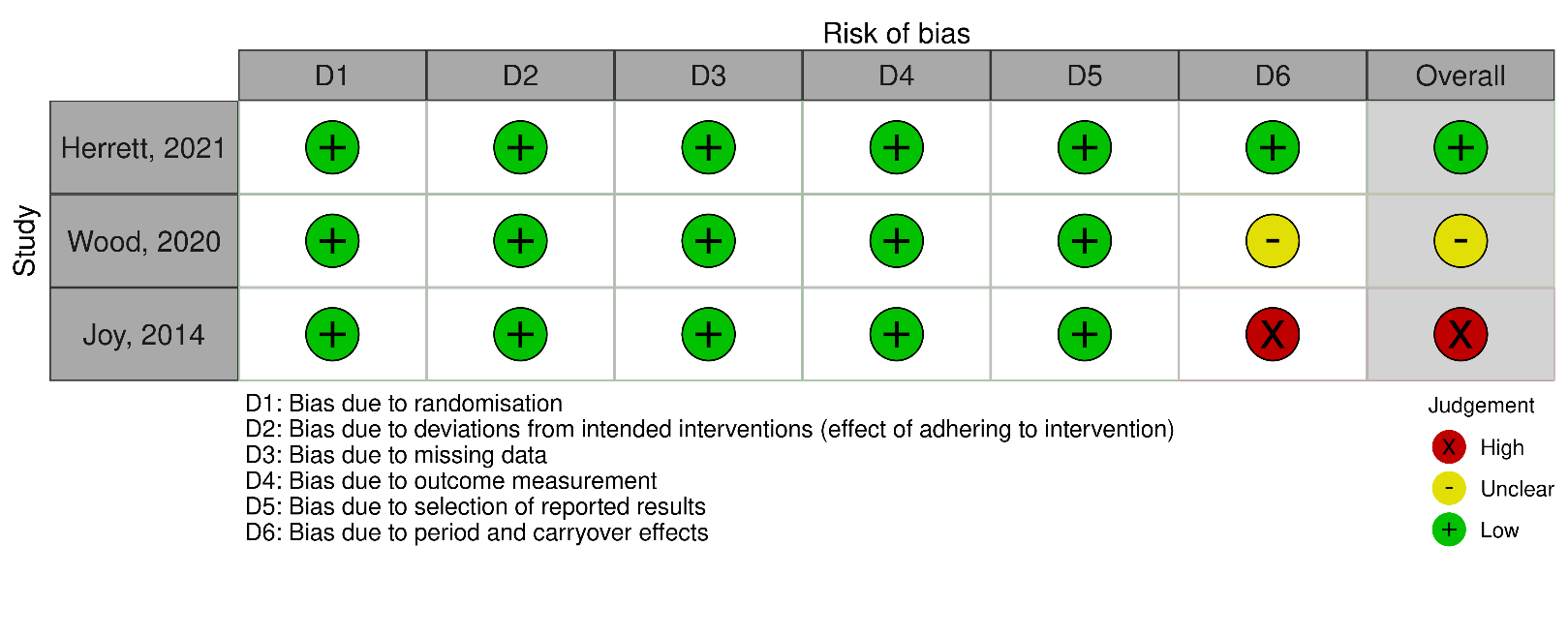 | | | | | | | | | |
| Risk of bias assessment of prospective studies with  ROBINS-I | | | | | | | | | |
| 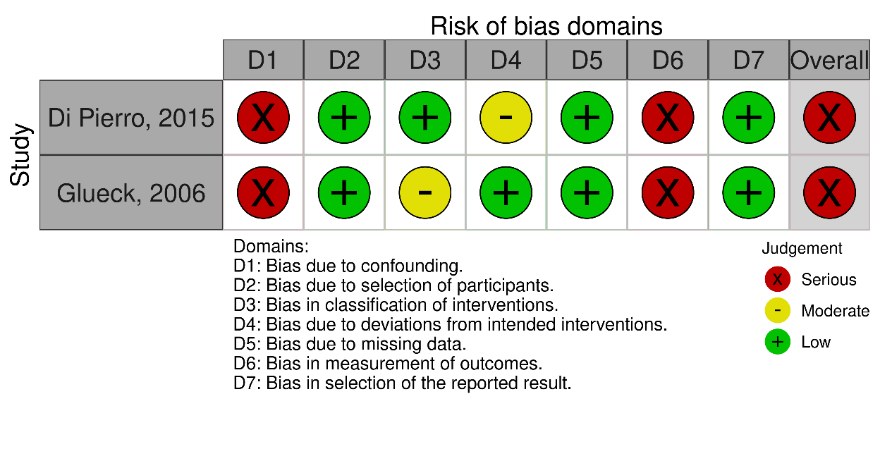 | | | | | | | | | |
| 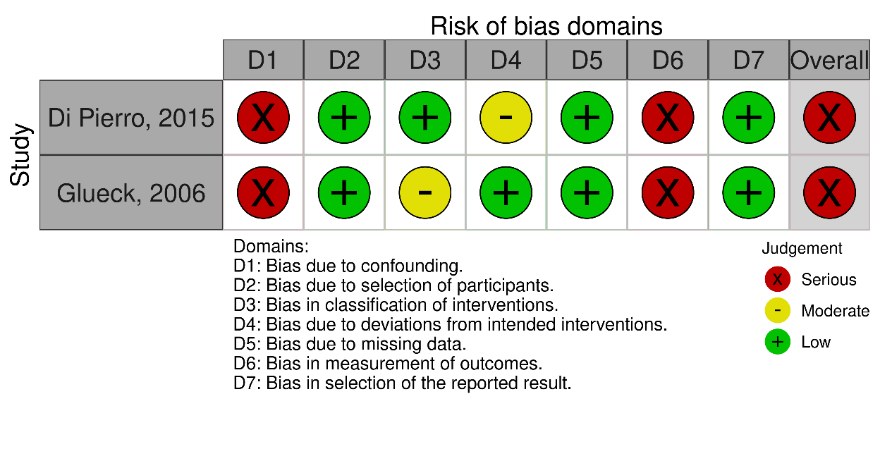 | | | | | | | | | |
| Risk of bias assessment of retrospective cohort studies with Newcastle-Ottawa Scale | | | | | | | | | |
|  | | Selection |  |  |  | Comparability | Outcome |  |  |
| Study | | ① | ② | ③ | ④ | ⑤ | ⑥ | ⑦ | ⑧ |
| Brennan | 2017 | ★ | ★ | ★ |  |  | ★ | ★ | ★ |
| Cicero | 2016 | ★ | ★ |  |  |  |  |  |  |
| Fung | 2012 | ★ | ★ | ★ |  |  | ★ | ★ |  |
| Harrison | 2018 | ★ | ★ | ★ |  |  | ★ | ★ | ★ |
| Kang | 2017 |  | ★ | ★ |  |  | ★ | ★ | ★ |
| Mampuya | 2013 | ★ | ★ | ★ |  | ★ | ★ | ★ | ★ |
| Meek | 2012 | ★ | ★ | ★ |  |  | ★ | ★ | ★ |
| Williams | 2015 | ★ | ★ | ★ |  |  | ★ | ★ | ★ |
| ①Representativeness of the exposed cohort; ②Selection of the non-exposed cohort; ③Ascertainment of exposure; ④Demonstration that the current outcome of interest was not present at start of study; ⑤Comparability of cohorts on the basis of the design or analysis; ⑥Assessment of outcome; ⑦Length of follow-up long enough for outcomes to occur; ⑧Adequacy of follow up of cohorts  ★ High quality choice | | | | | | | | | |
