# Supplementary material for: Safety outcomes of statin vs non-statin lipid-lowering interventions in patients with prior statin-associated muscle symptoms: A systematic review and meta-analysis
Source: PLoS One. 2025 Dec 11;20(12):e0338575. doi: 10.1371/journal.pone.0338575 (PMC12698018; doi:10.1371/journal.pone.0338575)
Supplement: S3 File — (DOCX) [file pone.0338575.s003.docx]

GRADE Table for included RCTs

| Outcome | Study, N | Risk of Bias  (RoB) | Imprecision | Inconsistency | Indirectness | Publication Bias | Quality of Evidence |
| --- | --- | --- | --- | --- | --- | --- | --- |
| Incidence of muscle symptoms | 9 RCT, 1569 | low to some concerns | neutral | neutral | neutral | low | moderate |
| Treatment discontinuation | 6 RCT, 781 | low to some concerns | neutral to -1 | neutral | neutral | low | moderate -low^1^ |
| Effectiveness | 3 RCT, 383 | low to some concerns | neutral to -1 | neutral | neutral to -1 | -1 | moderate -low^2^ |

1 due to some concerns in the risk of bias and imprecision assessment

2 due to some concerns in the risk of bias, imprecision, indirectness and publication bias assessment
