# Supplementary material for: Safety outcomes of statin vs non-statin lipid-lowering interventions in patients with prior statin-associated muscle symptoms: A systematic review and meta-analysis
Source: PLoS One. 2025 Dec 11;20(12):e0338575. doi: 10.1371/journal.pone.0338575 (PMC12698018; doi:10.1371/journal.pone.0338575)
Supplement: S4 File — (DOCX) [file pone.0338575.s004.docx]

S1 Supporting information 1: Tables of included studies

## Table 1: List of randomized controlled trials comparing statin-based therapy to a variety of comparators

| **Study, year, country/Continent** | **N** | **Intervention(s)** | **Comparator(s)** | **Exposure ‡** | **Assessment of muscle symptoms** | **Outcome assessments** | | **Age, mean (SD)** | **Male, n (%)** | **Definition of prior SAMS** |
| --- | --- | --- | --- | --- | --- | --- | --- | --- | --- | --- |
| **Parallel randomized controlled trials** | | | | | | |  |  |  |  |
| Wijekoon, 2020, Sri Lanka* | 101 | Atorvastatin  Non-daily dosing | Atorvastatin  Daily dosing | 24 wk | Composite of myalgia and myositis | 12 and 24 wk | | 61 (9.3) | 24 (24) | Self-reported SAMS |
| Moriarty, 2015,  North America, Europe | 314 | Atorvastatin | Alirocumab, Ezetimibe | 24 wk | Myalgia | 12 wk, 24 wk | | 63 (9.5) | 172 (55) | Self-reported SAMS with min. 2 statins |
| Pfizer, 2014, USA, unpublished | 184 | Atorvastatin | Placebo, Bococizumab | 24 wk | Myalgia | 12 wk, 24 wk | | 64 (8.5) | 85 (46) | Self-reported Intolerance to statins |
| Halbert, 2010, USA | 43 | Pravastatin | Red Yeast Rice | 12 wk | Combined local and generalized myalgia | 4, 8, 12 wk | | 63 (7.7) | 11 (25) | Self reported SAMS |
| Stein, 2008,DE | 199 | Fluvastatin + Ezetimibe | Ezetimibe | 12 wk | Muscle-related side effect | 12 wk | | 61 (10) | 95 (48) | Self reported SAMS |
| Howard, 2021 UK | 60 | Atorvastatin | Placebo, No therapy | 3x16 wk | Muscle symptom intensity | Daily, 6 months post trial | | 66 (8.6) | 35 (58) | Intolerance to statins |
| Kristiansen, 2020,NO | 71 | Atorvastatin | Placebo | 2x7 wk | Myalgia intensity (VAS) | Last 3 wk of period | | 64 (9.5) | 48 (68) | Self-reported SAMS |
| Taylor, 2015,USA | 120 | Simvastatin | Placebo | 2x8 wk | Myalgia intensity (BPI-SF) | 16 wk | | NR | 69 (58) | Self-reported SAMS |
| Kennedy, 2011,USA | 17 | Rosuvastatin | Placebo | 2x8 wk | Myalgia | 4, 8, 12,16 wk | | 66 (9.3) | 17 (100) | Self-reported SAMS |
| Nissen, 2016, AU/NZ | 491 | Atorvastatin | Placebo | 2x10 wk | Myalgia | 24 wk | | 61 (10.2) | 245 (50) | Self-reported SAMS |
| **N-of-1 Randomized controlled trials** | | |  |  |  |  | |  |  |  |
| Herrett, 2021, UK | 200 | Atorvastatin | Placebo | 6x 2 m. | Muscle symptom intensity | Every 2 months 12 months | | 69 (9.5) | 115 (58) | Self-reported SAMS |
| Wood, 2020,UK | 60 | Atorvastatin | Placebo | 12x 1 m. | Muscle symptom intensity | Daily during 12 months | | 66 (8.6) | 35 (58) | Discontinuation of Statin due to muscle symptoms |
| Joy, 2014, CA | 8 | Unspecified Statin | Placebo | 6x 3 wk. | Myalgia intensity (VAS, BPI-SF) | 7 ,14 , 21 d | | 66 (8) | 1 (12) | Self-reported SAMS without CK-Elevation |

**‡** If multiple exposure periods are observed, the trial was performed following a crossover design.

Abbreviations: N, number of participants; CI, confidence interval, m, month; NR, not reported; SD, Standard deviation; wk, weeks; AU, Australia; CA, Canada; DE, Germany; IT, Italy; NO, Norway; NZ, New Zealand; UK, United Kingdom; USA, United State of America; BPI-SF, Brief Pain Inventory Short Form; SAMS, Statin associated muscle symptoms; VAS, Visual analog score;

| **Study, year, country/Continent** | **N** | **Exposure(s)** | **Comparator(s)** | **Exposure** | **Assessment of muscle symptoms** | **Adjustment for covariates** | **Follow up** | **Age, mean (SD)** | **Male, n** | **Definition of prior SAMS** |
| --- | --- | --- | --- | --- | --- | --- | --- | --- | --- | --- |
| **Prospective studies** | | |  |  |  |  |  |  |  |  |
| Di Pierro, 2015,IT | ­45 | Unspecified statin + Berberin | Ezetimibe, Berberin | 12 m | Unspecific Intolerance | None | 12 m. | 67 (7) | 27 (60%) | Self-reported SAMS and elevation of CK |
| Glueck, 2006,USA | 61 | Rosuvastatin 5mg/d | Rosuvastatin 10mg/d | 5mg: 16 wk  10mg: 44 wk | Myalgia | Baseline LDL-C, age, BMI, treatment duration | 5mg: 16 wk  10mg: 44 wk | 60 (10) | 20 (33%) | Self-reported SAMS |
| **Retrospective studies** | | |  |  |  |  |  |  |  |  |
| Harrison,2018, USA | 45’037 | statin intensity up-titration, down-titration, same-statin rechallenge, statin switch**‡** | | 12 m. | Unspecific intolerance to statins | None | 12 m. | 63 (11.6) | 18'088 (40%) | Unspecific intolerance to statins |
| Brennan, 2017,USA | 118 | rechallenge, statin switch, non-daily dosing | Non-statin based therapy | 17 m. | Unspecific intolerance to statins | None | 17 m. | 67 (10.9) | 49 (42%) | Self-reported SAMS with min. 2 statins |
| Kang 2017, USA | 27 | Atorvastatin, Pravastatin, Rosuvastatin, Simvastatin, Lovastatin, Fluvastatin**‡** | | 12 m | Myalgia | None | 12 m. | 65 (8.9) | 26 (96%) | Self-reported SAMS with min. 2 statins |
| Cicero, 2016,IT | 252 | Rosuvastatin + Ezetemib | Fibrate + Ezetimibe,  Red Yeast Rice+Berberin+Ezetimibe, Berberine+Ezetimibe, Phytosterol+Psyllium+Ezetimibe | NR | Myalgia | None | NR | NR | 114 (45%) | Self-reported SAMS with min. 2 statins |
| Williams, 2015, UK | 50 | Statin+Ezetemib, Statin+Fibrate, Statin+Bile acid sequestrant, Statin+Omega-3 fatty acids | Ezetemib, Fibrate, Bile acid sequestrant, Omega-3 fatty acids | 10 m. | Unspecific intolerance to statins | None | 10 m. | 59 (NR) | 20 (40%) | Unspecific intolerance to statins |
| Mampuya, 2013,  CA | 1605 | Non-daily dosing  Daily Dosing | Non-statin based therapy | Median of 31m | Myalgia | Baseline lipid-parameter, other lipid-lowering drugs | median of 31 m. | 59 (NR) | 686 (42%) | Self-reported SAMS without CK-Elevation |
| Fung, 2012, UK | 108 | Atorvastatin, Rosuvastatin, Pravastatin, Fluvastatin | Fibrate, Colestyramine, Colesevelam, Ezetimibe | 18 m. | Myalgia | None | 18 m. | 58.3 (11.9) | 41 (38%) | Unclear |
| Meek, 2012, UK | 325 | Rosuvastatin 5mg/d, Rosuvastatin 5mg 2-3x/w, Rosuvastatin 5mg/w**‡** | | Median of 15m | Myalgia | None | median of 14.9 m. | 63 (10) | 159 (49%) | Self-reported SAMS without CK-Elevation |

## Table 2: List of prospective and retrospective cohort studies comparing statin-based therapy to a variety of comparators

**‡** The study only compared statin-based managements and had no non-statin comparator

Abbrevations: N, number of participants; CI, confidence interval, m, month; NR, not reported; SD, Standard deviation; wk, weeks; AU, Australia; CA, Canada; DE, Germany; IT, Italy; NO, Norway; NZ, New Zealand; UK, United Kingdom; USA, United State of America; SAMS, Statin associated muscle symptoms; BMI, Body Mass Index
