# Supplementary material for: Safety outcomes of statin vs non-statin lipid-lowering interventions in patients with prior statin-associated muscle symptoms: A systematic review and meta-analysis
Source: PLoS One. 2025 Dec 11;20(12):e0338575. doi: 10.1371/journal.pone.0338575 (PMC12698018; doi:10.1371/journal.pone.0338575)
Supplement: S5 File — (DOCX) [file pone.0338575.s005.docx]

Supporting information: Sensitivity analysis of the incidence of SAMS by removing Nissen et al.
