# Supplementary material for: Safety outcomes of statin vs non-statin lipid-lowering interventions in patients with prior statin-associated muscle symptoms: A systematic review and meta-analysis
Source: PLoS One. 2025 Dec 11;20(12):e0338575. doi: 10.1371/journal.pone.0338575 (PMC12698018; doi:10.1371/journal.pone.0338575)
Supplement: S6 File — (DOCX) [file pone.0338575.s006.docx]

Supporting information 6: Leave-on-out analysis on the incidence of muscle symptoms

| Study Left Out | Comparator | OR (95% - CI) | Heterogeneity I^2^ in % |
| --- | --- | --- | --- |
| Pfizer, 2014 | Statin versus Placebo or Statin & Ezetimibe versus Ezetimibe | 1.27 (0.7-2.3) | 51.2 |
| Nissen, 2016 | Statin versus Placebo or Statin & Ezetimibe versus Ezetimibe | 1.03 (0.65-1.61) | 14.8 |
| Halbert, 2010 | Statin versus Red Yeast Rice | 1.27 (0.77-2.08) | 48.3 |
| Stein, 2008 | Statin versus Ezetimibe | 1.27 (0.77-2.08) | 41.3 |
| Stein, 2008 | Statin versus Placebo or Statin & Ezetimibe versus Ezetimibe | 1.74 (1.34-2.26) | 33.8 |
| Moriarty, 2015 | Statin versus Ezetimibe | 1.27 (0.77-2.08) | 51.1 |
| Moriarty, 2015 | Statin versus PCSK9 Inhibitors | 1.27 (0.77-2.08) | 50.1 |
| Pfizer, 2014 | Statin versus Placebo or Statin & Ezetimibe versus Ezetimibe | 1.27 (0.77-2.08) | 51.3 |
| Wijekoon, 2020 | Statin daily dosing versus Statin non daily dosing versus | 1.27 (0.77-2.08) | 39.2 |
| Taylor, 2015 | Statin versus Placebo or Statin & Ezetimibe versus Ezetimibe | 1.18 (0.56-2.48) | 51.5 |
| Kennedy, 2011 | Statin versus Placebo or Statin & Ezetimibe versus Ezetimibe | 1.23 (0.71-2.13) | 51.7 |
