# Supplementary material for: Safety outcomes of statin vs non-statin lipid-lowering interventions in patients with prior statin-associated muscle symptoms: A systematic review and meta-analysis
Source: PLoS One. 2025 Dec 11;20(12):e0338575. doi: 10.1371/journal.pone.0338575 (PMC12698018; doi:10.1371/journal.pone.0338575)
Supplement: S7 File — (DOCX) [file pone.0338575.s007.docx]

Supporting information: Stratified analysis by statin-intensity

| **Outcome** | **Statin intensity** | **n (studies)** | **Pooled OR (95% CI)** | **I² (%)** |
| --- | --- | --- | --- | --- |
| **Incidence of muscle symptoms** | Low intensity | 9 | 1.46 (1.12 – 1.90) | 18.4 |
|  | Moderate–high intensity | 2 | 0.59 (0.32 – 1.08) | 0.0 |
| **Treatment discontinuation** | Low intensity | 5 | 1.78 (1.37 – 2.32) | 0.0 |
|  | Moderate–high intensity | 2 | 0.48 (0.16 – 1.44) | 0.0 |
