# Supplementary material for: Safety outcomes of statin vs non-statin lipid-lowering interventions in patients with prior statin-associated muscle symptoms: A systematic review and meta-analysis
Source: PLoS One. 2025 Dec 11;20(12):e0338575. doi: 10.1371/journal.pone.0338575 (PMC12698018; doi:10.1371/journal.pone.0338575)
Supplement: S8 File — (DOCX) [file pone.0338575.s008.docx]

Supporting information: List of included and excluded studies

Included Studies

RCT

1. Halbert, S.C., et al., *Tolerability of red yeast rice (2,400 mg twice daily) versus pravastatin (20 mg twice daily) in patients with previous statin intolerance.* American Journal of Cardiology, 2010. **105**(2): p. 198-204.
2. Herrett, E., et al., *Statin treatment and muscle symptoms: series of randomised, placebo controlled n-of-1 trials.* BMJ, 2021. **372**: p. n135.
3. Joy, T.R., et al., *N-of-1 (single-patient) trials for Statin-related Myalgia.* Annals of Internal Medicine, 2014. **160**(5): p. 301-310.
4. Kennedy, S.P., et al., *Efficacy and tolerability of once-weekly rosuvastatin in patients with previous statin intolerance.* Journal of Clinical Lipidology, 2011. **5**(4): p. 308-315.
5. Kristiansen, O., et al., *Effect of atorvastatin on muscle symptoms in coronary heart disease patients with self-perceived statin muscle side-effects: a randomized, double blinded crossover trial.* European Heart Journal Cardiovascular Pharmacotherapy, 2020. **01**: p. 01.
6. Moriarty, P.M., et al., Efficacy and safety of alirocumab vs ezetimibe in statin-intolerant patients, with a statin rechallenge arm: the ODYSSEY ALTERNATIVE randomized trial. Journal of clinical lipidology, 2015. 9(6): p. 758‐769.
7. Nissen, S.E., et al., *Efficacy and Tolerability of Evolocumab vs Ezetimibe in Patients With Muscle-Related Statin Intolerance: the GAUSS-3 Randomized Clinical Trial.* JAMA, 2016. **315**(15): p. 1580‐1590.
8. Pfizer, Randomized Clinical Trial of Bococizumab (PF-04950615; RN316) in Subjects With Primary Hyperlipidemia or Mixed Dyslipidemia At Risk Of Cardiovascular Events. 2014, https://ClinicalTrials.gov/show/NCT02100514.
9. Stein, E.A., et al., *Efficacy and tolerability of fluvastatin XL 80 mg alone, ezetimibe alone, and the combination of fluvastatin XL 80 mg with ezetimibe in patients with a history of muscle-related side effects with other statins.* American Journal of Cardiology, 2008. **101**(4): p. 490-6.
10. Taylor, B.A., et al., *A randomized trial of coenzyme Q10 in patients with confirmed statin myopathy.* Atherosclerosis, 2015. **238**(2): p. 329‐335.
11. Wood, F.A., et al., *N-of-1 Trial of a Statin, Placebo, or No Treatment to Assess Side Effects.* N Engl J Med, 2020. **383**(22): p. 2182-2184.
12. Wijekoon, N., et al., Tolerability and effectiveness of every-other-day atorvastatin compared to daily atorvastatin in patients with muscle symptoms: A randomized controlled clinical trial. Contemporary Clinical Trials Communications, 2020. 20.
13. Slctr, *Tolerability and effectiveness of every-other-day atorvastatin dosing in patients with statin related muscle disease.* <http://www.who.int/trialsearch/Trial2.aspx?TrialID=SLCTR/2015/010>, 2015.

**Prospective studies**

1. Di Pierro, F., et al., *Clinical role of a fixed combination of standardized Berberis aristata and Silybum marianum extracts in diabetic and hypercholesterolemic patients intolerant to statins.* Diabetes, Metabolic Syndrome and Obesity: Targets and Therapy, 2015. **8**: p. 89-96.
2. Glueck, C.J., et al., *Rosuvastatin 5 and 10 mg/d: A pilot study of the effects in hypercholesterolemic adults unable to tolerate other statins and reach ldl cholesterol goals with nonstatin lipid-lowering therapies.* Clinical Therapeutics, 2006. **28**(6): p. 933-942.

**Retrospective studies**

1. Brennan, E.T. and T.R. Joy, *Management Strategies for Statin-Associated Muscle Symptoms: How Useful Is Same-Statin Rechallenge?* Canadian Journal of Cardiology, 2017. **33**(5): p. 666-673.
2. Cicero, A.F.G., et al., *Additional therapy for cholesterol lowering in ezetimibe-treated, statin-intolerant patients in clinical practice: results from an internal audit of a university lipid clinic.* Current Medical Research and Opinion, 2016. **32**(10): p. 1633-1638.
3. Fung, E.C. and M.A. Crook, *Statin myopathy: A lipid clinic experience on the tolerability of statin Rechallenge.* Cardiovascular Therapeutics, 2012. **30**(5): p. e212-e218.
4. Harrison, T.N., et al., Unmet Patient Need in Statin Intolerance: the Clinical Characteristics and Management. Cardiovascular Drugs & Therapy, 2018. 32(1): p. 29-36.
5. Kang, J.H., et al., *Rechallenging Statin Therapy in Veterans with Statin-Induced Myopathy Post Vitamin D Replenishment.* Journal of Pharmacy Practice, 2017. **30**(5): p. 521-527.
6. Mampuya, W.M., et al., *Treatment strategies in patients with statin intolerance: The Cleveland Clinic experience.* American Heart Journal, 2013. **166**(3): p. 597-603.
7. Meek, C., et al., Daily and intermittent rosuvastatin 5mg therapy in statin intolerant patients: An observational study. Current Medical Research and Opinion, 2012. 28(3): p. 371-378.
8. Williams, K. and V. Mishra, *The Impact of Statin Intolerance in Lipid Clinic Patients.* International Journal of Clinical Medicine, 2015. **06**: p. 314-321.

**Excluded records**

1. Abourjaily, 2003

Abourjaily, H.M., A.A. Alsheikh-Ali, and R.H. Karas, Comparison of the frequency of adverse events in patients treated with atorvastatin or simvastatin. American Journal of Cardiology, 2003. 91(8): p. 999-1002.

Reason for exclusion: unsuitable population (evaluation of database about adverse events)

1. Athyros, 2008

Athyros, V.G., et al., Effectiveness of ezetimibe alone or in combination with twice a week Atorvastatin (10 mg) for statin intolerant high-risk patients. American Journal of Cardiology, 2008. 101(4): p. 483-5.

Reason for exclusion: unsuitable intervention.

1. Backes, 2007

Backes, J.M., et al., Effects of once weekly rosuvastatin among patients with a prior statin intolerance. American Journal of Cardiology, 2007. 100(3): p. 554-5.

Reason for exclusion:no comparator arm.

1. Ballantyne, 2018

Ballantyne, C.M., et al., Phase 3 evaluation of bempedoic acid added to ezetimibe in patients with elevated LDL-cholesterol receiving no greater than low dose statins: Clear tranquility. Atherosclerosis Supplements, 2018. 32: p. 25.

Reason for exclusion: Abstract

1. Ballantyne, 2018

Ballantyne, C.M., et al., Efficacy and safety of bempedoic acid added to ezetimibe in statin-intolerant patients with hypercholesterolemia: A randomized, placebo-controlled study. Atherosclerosis, 2018. 277: p. 195-203.

Reason for exclusion:Co-intervention only in one arm.

1. Birg, 2013

Birg, A., et al., Vitamin D levels in statin intolerant patients and effect of vitamin D repletion on successful statin rechallenge. Journal of Investigative Medicine, 2013. 61(2): p. 396-397.

Reason for exclusion:Conference paper

1. Blaier, 2011

Blaier, O., M. Lishner, and A. Elis, Managing statin-induced muscle toxicity in a lipid clinic. Journal of Clinical Pharmacy & Therapeutics, 2011. 36(3): p. 336-41.

Reason for exclusion:no comparator arm.

1. Bosgrud, 2009

Bogsrud, M., et al., The effect of q10 and selenium supplement on adverse effects in statin treatment. Atherosclerosis Supplements, 2009. 10(2).

Reason for exclusion:Abstract

1. Bosgrud, 2013

Bogsrud, M.P., et al., No effect of combined coenzyme Q10 and selenium supplementation on atorvastatin-induced myopathy. Scandinavian Cardiovascular Journal, 2013. 47(2): p. 80-7.

Reason for exclusion:Co-intervention only in one arm.

1. Bookstaver, 2011

Bookstaver, D.A. and N.A. Burkhalter, Effect of CoEnzyme Q10 supplementation on HMG-CoA reductase inhibitor-induced myalgias. Pharmacotherapy, 2011. 31(10): p. 343e-344e.

Reason for exclusion:Co-intervention only in one arm.

1. Brennan, 2013

Brennan, E.T. and T. Joy, Efficacy of differing lipid-lowering management strategies in patients with a history of statin-related myopathy. Endocrine Reviews, 2013. 34(3).

Reason for exclusion:Conference abstract

1. Buettner, 2009

Buettner, C., et al., Clinical Trial of CoQ10 for Mild-to-Moderate Statin-Associated Muscle Symptoms. 2009, https://ClinicalTrials.gov/show/NCT01032993.

Reason for exclusion: Co-intervention only in one arm.

1. Buettner, 2015

Buettner, C., et al., COQ10 does not improve statin myalgiaea randomized controlled trial. Atherosclerosis, 2015. 241(1): p. e206.

Reason for exclusion: Conference abstract.

1. Center, 2012

Center, C.-S.M., Is Treatment of Vitamin D Deficiency Associated With Resolution of Statin-Induced Muscular Symptoms. 2012, https://ClinicalTrials.gov/show/NCT01568255.

Reason for exclusion: abandoned study. Not enough information

1. Cham, 2010

Cham, S., et al., Statin-associated muscle-related adverse effects: a case series of 354 patients. Pharmacotherapy:The Journal of Human Pharmacology & Drug Therapy, 2010. 30(6): p. 541-53.

Reason for exclusion: no comparator arm.

1. Cha, 2023

Cha JJ, Hong SJ, Kim JH, Lim S, Joo HJ, Park JH, Yu CW, Lee PH, Lee SW, Lee CW, Moon JY, Lee JY, Kim JS, Park JS, Lee K, Lim SY, Na JO, Cho JM, Kim SY, Lim DS. Effect of rosuvastatin 20 mg versus rosuvastatin 5 mg plus ezetimibe on statin side-effects in elderly patients with atherosclerotic cardiovascular disease: Rationale and design of a randomized, controlled SaveSAMS trial. Am Heart J. 2023 Jul;261:45-50. doi: 10.1016/j.ahj.2023.03.002. Epub 2023 Mar 17. PMID: 36934981.

Reason for exclusion: no non-statin comparator

1. Cheeley, 2015

Cheeley, M.K., C. Ni, and T.A. Jacobson, Incidence of statin intolerance in a referral lipid clinic: Patient characteristics and response to rechallenge. Journal of Clinical Lipidology, 2015. 9(3): p. 461-462.

Reason for exclusion:no comparator arm.

1. Cho, 2014

Cho, L., et al., Clinical profile of statin intolerance in the phase 3 gauss-2 study. Canadian Journal of Cardiology, 2014. 30(10): p. S79.

Reason for exclusion: Abstract of Cho, 2016

1. Cho, 2016

Cho, L., et al., Clinical Profile of Statin Intolerance in the Phase 3 GAUSS-2 Study. Cardiovascular Drugs & Therapy, 2016. 30(3): p. 297-304.

Reason for exclusion:Report of the characteristics ot the population enrolled in the phase 3 Goal Achievement after Utilizing ananti-PCSK9 antibody in StatinIntolerant Subjects Study(GAUSS-2; NCT 01763905).

1. Choi, 2023

Choi H, Kang SH, Jeong SW, Yoon CH, Youn TJ, Song WH, Jeon DW, Lim SW, Lee JH, Cho SW, Chae IH, Kim CH. Lipid-Lowering Efficacy of Combination Therapy With Moderate-Intensity Statin and Ezetimibe Versus High-Intensity Statin Monotherapy: A Randomized, Open-Label, Non-Inferiority Trial From Korea. J Lipid Atheroscler. 2023 Sep;12(3):277-289. doi: 10.12997/jla.2023.12.3.277. Epub 2023 Aug 3. PMID: 37800112; PMCID: PMC10548190.

Reason for exclusion: no non-statin comparator

1. Cicero, 2018

Cicero, A.F.G., et al., An easy strategy to manage night cramps associated to statin assumption: Results from a real practice study. Journal of Hypertension, 2018. 36: p. e40-e41.

Reason for exclusion: Abstract

1. Clark, 2016

Clark, D., et al., Clinical characteristics associated with complete statin intolerance: The Cleveland clinic experience. Journal of the American College of Cardiology, 2016. 67(13): p. 1895.

Reason for exclusion: Conference abstract of Cleveland Clinic Experience

1. Compton, 2020

Compton, A., et al., Success rate of statin rechallenge after the initiation of vitamin D supplementation in statin intolerant patients. JACCP Journal of the American College of Clinical Pharmacy, 2020. 3(1): p. 158.

Reason for exclusion:Co-intervention only in one arm.

1. De Flaviis, 2019

De Flaviis, C., et al., Statin-Induced Myopathy: Different Strategies For Management And Difficult Challenges To Reduce Cardiovascular Risk. Atherosclerosis, 2019. 287: p. e202.

Reason for exclusion: unsuitable intervention.Co-intervention only in one arm.

1. Degreef, 2010

Degreef, L.E., et al., The tolerability and efficacy of low-dose simvastatin in statin-intolerant patients. European Journal of Internal Medicine, 2010. 21(4): p. 293-6.$

Reason for exclusion: no comparator arm.

1. Derosa, 2019

Derosa, G., A. D'Angelo, and P. Maffioli, Coenzyme q10 liquid supplementation in dyslipidemic subjects with statin-related clinical symptoms: a double-blind, randomized, placebo-controlled study. Drug design, development and therapy, 2019. 13: p. 3647‐3655.

Reason for exclusion: Co-intervention only in one arm.

1. Derosa, 2015

Derosa, G., et al., Berberis aristata combined with Silybum marianum on lipid profile in patients not tolerating statins at high doses. Atherosclerosis, 2015. 239(1): p. 87-92.

Reason for exclusion: Co-intervention only in one arm.

1. Derosa, 2015

Derosa, G., et al., Berberis aristata/Silybum marianum fixed combination (Berberol®) effects on lipid profile in dyslipidemic patients intolerant to statins at high dosages: A randomized, placebo-controlled, clinical trial. Phytomedicine, 2015. 22(2): p. 231-237.

Reason for exclusion: Co-intervention only in one arm

1. Fayfman, 2015

Fayfman, M., et al., Long-term efficacy of non-daily statin dosing in previously intolerant patients: The Atlanta va medical center experience. Journal of Clinical Lipidology, 2015. 9(3): p. 460-461.

Reason for exclusion: Conference abstract. No comparator.

1. Farnier, 2024

Farnier, M. et al. Simulation of ezetimibe and Bempedoic acid effect on LDL-C goal attainment in statin-intolerant patients across Europe: The 1-year follow-up Santorini observational study; Atherosclerosis, Volume 395, 118176; DOI: [10.1016/j.atherosclerosis.2024.118176](https://doi.org/10.1016/j.atherosclerosis.2024.118176)

Reason for exclusion: Wrong study design

1. Fedacko, 2013

Fedacko, J., et al., Coenzyme Q(10) and selenium in statin-associated myopathy treatment. Canadian Journal of Physiology & Pharmacology, 2013. 91(2): p. 165-70.

Reason for exclusion: Co-intervention only in one arm.

1. Fernando 2024

Fernando K. et al.; Efficacy, Safety and Cost-effectiveness of Atorvastatin 40mg versus 80 mg in South Asian Patients with acute coronary syndrome: A protocol for randomised clinical trial

MedRxiv 2024.08.09.24311739; doi: https://doi.org/10.1101/2024.08.09.24311739

Reason for exclusion: Protocol

1. Gadarla, 2008

Gadarla, M., A.K. Kearns, and P.D. Thompson, Efficacy of rosuvastatin (5 mg and 10 mg) twice a week in patients intolerant to daily statins. American Journal of Cardiology, 2008. 101(12): p. 1747-8.

Reason for exclusion:no comparator arm.

1. Gargiulo, 2023

Gargiulo P, Basile C, Cesaro A, Marzano F, Buonocore D, Asile G, Abbate V, Vicidomini F, Paolillo S, Spaccarotella CAM, Catalano A, Spirito G, Merlini PA, Maloberti A, Iannuzzo G, Ciccone MM, Zito AP, Paloscia L, D'Alleva A, Varbella F, Corleto A, Brunetti ND, Corbo MD, Calabrò P, Indolfi C, Perrone-Filardi P. Efficacy, safety, adherence and persistence of PCSK9 inhibitors in clinical practice: A single country, multicenter, observational study (AT-TARGET-IT). Atherosclerosis. 2023 Feb;366:32-39. doi: 10.1016/j.atherosclerosis.2023.01.001. Epub 2023 Jan 13. PMID: 36696749.

Reason for exclusion: Not specified if previous SAMS

1. Gazzi, 2018

Gazzi, L., et al., LIPID MODIFYING THERAPY AND LDL-C ACHIEVEMENTS IN A VERY HIGH CARDIOVASCULAR RISK COHORT OF STATIN INTOLERANT PATIENTS MANAGED BY GENERAL PRACTITIONERS IN ITALY. Value in Health, 2018. 21: p. S96.

Reason for exclusion: Abstract. No comparator.

1. Giladi 2024

Giladi E, Israel R, Daud W, Gurevitz C, Atamna A, Pereg D, Assali A, Elis A. Anti PCSK9 Monoclonal Antibody Treatment in Elderly Patients: A Real-world Clinical Experience. Isr Med Assoc J. 2024 Feb;26(2):130-135. PMID: 38420988.

Reason for exlusion: non statin group

1. Glueck, 2011

Glueck, C.J., et al., Vitamin D deficiency, myositis-myalgia, and reversible statin intolerance. Current Medical Research & Opinion, 2011. 27(9): p. 1683-90.

Reason for exclusion: no comparator arm.

1. Goldberg, 2012

Goldberg, A.S., et al., Rosuvastatin drug levels in non-daily rosuvastatin regimen. Canadian Journal of Diabetes, 2012. 36(5): p. S9.

Reason for exclusion: Abstract

1. Goldberg, 2013

Goldberg, A.S., et al., Efficacy and plasma drug concentrations with nondaily dosing of rosuvastatin. Canadian Journal of Cardiology, 2013. 29(8): p. 915-9.

Reason for exclusion: no outcome of interest. Only assessment of LDL in non daily treatement without comparison with daily traitement. Tolerability only in non daily treatment also.

1. Israeli, 1989

Israeli, A., et al., LOVASTATIN AND ELEVATED CREATINE KINASE: RESULTS OF RECHALLENGE. The Lancet, 1989. 333(8640): p. 725.

Reason for exclusion:Case reports of 3 patients with rechallenge from a RCT. No comparator.

1. Kao, 2019

Kao, C., et al., Review of Statin-Associated Symptoms, Definition and Diagnosis, and Treatment Strategies. An Australian Lipid Clinic experience. Heart Lung and Circulation, 2019. 28: p. S318.

Reason for exclusion:Abstract. No comparator.

1. Kok, 2017

Kok, C., et al., N-of-1 trials for myalgia in people taking a statin. Heart Lung and Circulation, 2017. 26: p. S120.

Reason for exclusion: Abstract of an included study (Joy, 2014)

1. Kraut 2023

Kraut R, Wierenga F, Molstad E, Korownyk C, Perry D, Dennett L, Garrison S. Intolerance upon statin rechallenge: A systematic review and meta-analysis of randomized controlled trials. PLoS One. 2023 Dec 21;18(12):e0295857. doi: 10.1371/journal.pone.0295857. PMID: 38128013; PMCID: PMC10735036.

Reason for exclusion: Wrong study design

1. Lakey, 2016

Lakey WC, Greyshock NG, Kelley CE, Siddiqui MA, Ahmad U, Lokhnygina YV, Guyton JR. Statin intolerance in a referral lipid clinic. J Clin Lipidol. 2016 Jul-Aug;10(4):870-879.e3. doi: 10.1016/j.jacl.2016.03.004. Epub 2016 Mar 17. PMID: 27578118.

Reason for exclusion: non non-statin comparator

1. Laufs, 2018

Laufs, U., et al., Efficacy and safety of bempedoic acid in patients with hypercholesterolemia and statin intolerance. Circulation, 2018. 138(25): p. e766.

Reason for exclusion:Abstract

1. Laufs, 2019

Laufs, U., et al., Efficacy and Safety of Bempedoic Acid in Patients With Hypercholesterolemia and Statin Intolerance. J Am Heart Assoc, 2019. 8(7): p. e011662.

Reason for exclusion:Co-intervention only in one arm.

1. Lee. 2023

Lee SJ, Cha JJ, Choi WG, Lee WS, Jeong JO, Choi S, Cho YH, Park W, Yoon CH, Lee YJ, Hong SJ, Ahn CM, Kim BK, Ko YG, Choi D, Hong MK, Jang Y, Hong SJ, Kim JS; RACING Investigators. Moderate-Intensity Statin With Ezetimibe Combination Therapy vs High-Intensity Statin Monotherapy in Patients at Very High Risk of Atherosclerotic Cardiovascular Disease: A Post Hoc Analysis From the RACING Randomized Clinical Trial. JAMA Cardiol. 2023 Sep 1;8(9):853-858. doi: 10.1001/jamacardio.2023.2222. Erratum in: JAMA Cardiol. 2023 Sep 1;8(9):891. doi: 10.1001/jamacardio.2023.3311. PMID: 37531130; PMCID: PMC10398545.

Reason for exclusion: wrong comparator

1. Lu, 2011

Lu, Z., et al., High long term success rates for statin rechallenge in patients referred for statin intolerance. Heart Lung and Circulation, 2011. 20: p. S53.

Reason for exclusion:Conference abstract. No comparator.

1. Maierhofer, 2011

Maierhofer, S., et al., Effectivity of colesevelam in statin intolerant patients ± ezetimibe. Atherosclerosis Supplements, 2011. 12(1): p. 163.

Reason for exclusion:Abstract. No statin treatment.

1. Mampuya, 2011

Mampuya, W., et al., Outcomes in statin intolerant patients: The cleveland clinic experience. Circulation, 2011. 124(21).

Reason for exclusion: Abstract of Mampuya, 2013

1. Marazzi, 2017

Marazzi, G., et al., Comparison of Low-Dose Statin Versus Low-Dose Statin + Armolipid Plus in High-Intensity Statin-Intolerant Patients With a Previous Coronary Event and Percutaneous Coronary Intervention (ADHERENCE Trial). American Journal of Cardiology, 2017. 120(6): p. 893-897.

Reason for exclusion: Co-intervention only in one arm.

1. Marazzi, 2014

Marazzi, G., et al., Randomized trial of the association between low dose statins and nutraceuticals in high intensity statin. Intolerrant patients with very high risk coronary artery disease (ADHERENCE). Circulation, 2014. 130.

Reason for exclusion: Abstract of Marazzi, 2017

1. Miadema 2023

Miedema MD, Gamam A, Garberich R, White S, Benson G. A Double-Blinded Randomized N-of-1 Trial to Facilitate Tolerance of Unblinded Rosuvastatin: The DESIFOR Pilot Trial. JACC Adv. 2023 Sep 26;2(8):100628. doi: 10.1016/j.jacadv.2023.100628. PMID: 38938347; PMCID: PMC11198235.

Reason for exclusion: Co-intervention with ezetimib and PCSK-9 inhibitors

1. Minissian, 2015

Minissian, M., et al., Do women with statin-related myalgias have low vitamin D levels? BMC Research Notes, 2015. 8: p. 449.

Reason for exclusion:not outcome of interest.

1. Minissian, 2012

Minissian, M., et al., Do women with statin-related myalgias have low vitamin D levels? Journal of the American College of Cardiology, 2012. 59(13): p. E1909.

Reason for exclusion: Abstract of Minissian, 2015

1. Mizeracki, 2012

Mizeracki, A.M. and M. Elam, Hypovitaminosis d and statin intolerance-utility of vitamin d repletion. Journal of Investigative Medicine, 2012. 60(1): p. 449.

Reason for exclusion:Conference Absract of an non-english study.

1. Moriarty, 2020

Moriarty, P.M., et al., Efficacy and safety of alirocumab in statin-intolerant patients over 3 years: open-label treatment period of the ODYSSEY ALTERNATIVE trial. Journal of Clinical Lipidology, 2020. 14(1): p. 88-97.e2.

Reason for exclusion: no statin use.

1. Munkhaugen, 2019

Munkhaugen, J., et al., Statin-associated muscle symptoms in coronary patients: design of a randomized study. Scandinavian Cardiovascular Journal, 2019. 53(3): p. 162-168.

Reason for exclusion: Protocol

1. Nair, 2008

Nair, R.K., R.L. Karadi, and E.S. Kilpatrick, Managing patients with 'statin intolerance': A retrospective study. British Journal of Cardiology, 2008. 15(3): p. 158-160.

Reason for exclusion: no information about the distribution of participants in the different intervention. no description of reason for discontinuation.

1. Norwitz, 2024

Norwitz NG, Cromwell WC. Oreo Cookie Treatment Lowers LDL Cholesterol More Than High-Intensity Statin therapy in a Lean Mass Hyper-Responder on a Ketogenic Diet: A Curious Crossover Experiment. Metabolites. 2024 Jan 22;14(1):73. doi: 10.3390/metabo14010073. PMID: 38276308; PMCID: PMC10818743.

Reason for exclusion: Wrong outcome

1. Nguyen, 2019

Nguyen, H., A. Thomasian, and T. Jaghasspanian, Evaluation of providers' response to statin re-challenge intervention. Journal of Managed Care and Specialty Pharmacy, 2019. 25: p. S91.

Reason for exclusion: Conference abstract about pharmacist calling provider to re-challeng patient.

1. Parker, 2013

Parker, B.A., et al., A randomized trial of coenzyme Q10 in patients with statin myopathy: rationale and study design. Journal of Clinical Lipidology, 2013. 7(3): p. 187-93.

Reason for exclusion:Rationale and study design of included study (Taylor, 2015)

1. Peyser, 2018

Peyser, B., et al., Effects of Delivering SLCO1B1 Pharmacogenetic Information in Randomized Trial and Observational Settings. Circulation. Genomic and Precision Medicine, 2018. 11(9): p. e002228.

Reason for exclusion: not intervetion of interest. (Genotype Informed Statin therapy vs usual care. No assessment of tolerability or acceptability.

1. Reddy, 2009

Reddy, K.J., et al., Efficacy of combination drug pulse therapy in maintaining lipid levels in patients intolerant of daily statin use. Journal of Clinical Hypertension, 2009. 11(12): p. 766-8.

Reason for exclusion:no outcome of interest.

1. Ruisinger, 2009

Ruisinger, J.F., et al., Once-a-week rosuvastatin (2.5 to 20 mg) in patients with a previous statin intolerance. American Journal of Cardiology, 2009. 103(3): p. 393-4.

Reason for exclusion:no comparator arm.

1. Sbrana, 2017

Sbrana, F., et al., Statin intolerance in heterozygous familial hypercolesterolemia with cardiovascular disease: After PCSK-9 antibodies what else? European Journal of Preventive Cardiology, 2017. 24(14): p. 1528-1531.

Reason for exclusion:no comparator arm.

1. Sinzinger, 2001

Sinzinger, H., et al., Isoprostane 8-epi-PGF2alpha is frequently increased in patients with muscle pain and/or CK-elevation after HMG-Co-enzyme-A-reductase inhibitor therapy. Journal of Clinical Pharmacy & Therapeutics, 2001. 26(4): p. 303-10.

Reason for exclusion: no outcome of interest.

1. Sirtori, 1989

Sirtori, C.R., et al., Clinical evaluation of simvastatin in patients with severe hypercholesterolemia. An Italian open study. Current Therapeutic Research - Clinical and Experimental, 1989. 46(2): p. 230-239.

Reason for exclusion: Translated Abstract. .

1. Snejdrlova, 2020

Snejdrlova, M., et al., Statin Intolerance in Clinical Practice. Current Atherosclerosis Reports, 2020. 22(7): p. 27.

Reason for exclusion: no comparator arm.

1. Stein, 2013

Stein, E.A., et al., Efficacy and tolerability of long-term treatment with AMG 145 in patients with statin intolerance. Circulation, 2013. 128(22).

Reason for exclusion: Abstract

1. Sullivan, 2012

Sullivan, D., et al., Effect of a monoclonal antibody to PCSK9 on low-density lipoprotein cholesterol levels in statin-intolerant patients: the GAUSS randomized trial. JAMA, 2012. 308(23): p. 2497-506.

Reason for exclusion:only 16% of the population used statins at baseline.

1. Taylor, 2014

Taylor, B., et al., Coenzyme Q10 does not reduce muscle pain in patients with confirmed statin Myalgia. Circulation, 2014. 130.

Reason for exclusion: Abstract of included study (Taylor, 2015)

1. University, Rockfeller., 2009

University, R., C. University, and A. University, Quality of Life in Patients With Statin-Associated Myopathy. 2009, https://ClinicalTrials.gov/show/NCT00850460.

Reason for exclusion: abandoned study. Not enough information

1. Vidyarthi, 2011

Vidyarthi, M. and T.A. Chowdhury, The effect of alternative dosing of rosuvastatin combined with daily coenzyme Q10 supplementation in patients previously intolerant to statins. Journal of Diabetes, 2011. 3: p. 196.

Reason for exclusion: Conference abstract of Vidyarthri, 2012 with only 19 patients

1. Vidyarthi, 2012

Vidyarthi, M., P. Jacob, and T.A. Chowdhury, Oral use of "Low and Slow" Rosuvastatin with Co-Enzyme Q10 in patients with Statin-Induced Myalgia: Retrospective case review. Indian Journal of Endocrinology and Metabolism, 2012. 16(Suppl 2): p. S498-500.

Reason for exclusion: no comparator arm.

1. Visser, 2011

Visser, M., et al., A randomized, double-blind, placebo-controlled trial to evaluate the effect of weekly subcutaneous injections of mipomersen, an apolipoprotein B-100 synthesis inhibitor, on low density lipoprotein cholesterol in high-risk statin-intolerant patients with hypercholesterolemia. Circulation, 2011. 124(21).

Reason for exclusion:Abstract of Visser, 2012

1. Visser, 2012

Visser, M.E., et al., Mipomersen, an apolipoprotein B synthesis inhibitor, lowers low-density lipoprotein cholesterol in high-risk statin-intolerant patients: a randomized, double-blind, placebo-controlled trial. European Heart Journal, 2012. 33(9): p. 1142-9.

Reason for exclusion:no statins treatment.

1. Voora, 2016

Voora, D., et al., SLCO1B1 genotype-guided statin therapy lowers LDL cholesterol in patients with statin-intolerance-a randomized controlled trial. Circulation, 2016. 134.

Reason for exclusion: Abstract of included study

1. Wierzbicki, 2005

Wierzbicki, A.S., et al., Efficacy of ezetimibe in patients with statin-resistant and statin-intolerant familial hyperlipidaemias. Current Medical Research & Opinion, 2005. 21(3): p. 333-8.

Reason for exclusion:16% of intolerant without statin treatment.

1. Wierzbicke, 2011

Wierzbicki, A.S., et al., The efficacy and safety of rosuvastatin 5mg in statin intolerant patients: An observational study. Journal of Clinical Lipidology, 2011. 5(3): p. 235-236.

Reason for exclusion:Abstract of Meek, 2012)

1. Young, 2007

Young, J.M., et al., Effect of coenzyme Q(10) supplementation on simvastatin-induced myalgia. American Journal of Cardiology, 2007. 100(9): p. 1400-3.

Reason for exclusion:Co-intervention only in one arm.

1. Zhang, 2013

Zhang, H., et al., Discontinuation of statins in routine care settings: a cohort study. Annals of Internal Medicine, 2013. 158(7): p. 526-34.

Reason for exclusion:no statins re-challenge.

1. Zhang, 2014

Zhang, H., et al., Adverse reactions to statins and discontinuation of statin therapy in patients with diabetes in routine care settings. Diabetes, 2014. 63: p. A157.

Reason for exclusion: Abstract of Zhang, 2013
